# Dietary variability in Middle Holocene South American shellmounds: Insights from isotopic analysis and an adapted Bayesian MixSIAR model

**DOI:** 10.1371/journal.pone.0335680

**Published:** 2025-12-03

**Authors:** Marina Di Giusto, André Fumis, Murilo Bastos, Victor Fossaluza, Rafael Stern, Jéssica Mendes Cardoso, Estelle Herrscher, Klervia Jaouen, Veronica Wesolowski

**Affiliations:** 1 CNRS, Géosciences Environnement Toulouse, Observatoire Midi Pyrénées, Toulouse, France,; 2 Museum of Archaeology and Ethnology, University of São Paulo, São Paulo, Brazil; 3 Institut of Mathematics and Statistics, University of São Paulo, São Paulo, Brazil; 4 National Museum, Federal University of Rio de Janeiro, Rio de Janeiro, Brazil; 5 Austrian Archaeological Institute, Austrian Academy of Sciences, Vienna, Austria; 6 CNRS, Aix-Marseille Université, Ministère de la Culture, LAMPEA, Aix-en-Provence, France; University of Modena and Reggio Emilia: Universita degli Studi di Modena e Reggio Emilia, ITALY

## Abstract

The degree of homogeneity in the diet of archaeological populations associated with Brazilian shellmounds (*sambaquis*) is an ongoing debate. Isotopic studies have the potential to document both intra- and inter-group dietary variability, especially when paired with quantitative tools, such as Bayesian Mixing Models. In this study, we investigate intra-site variability on diet at two shellmounds in southeastern Brazil: Piaçaguera (7151–5668 yBP), characterized by two distinct burial clusters, and Moraes (6791–5590 yBP). We analyzed *δ*^13^C and *δ*^15^N on human and fauna bone collagen (*n* = 43) and on human dentine collagen (*n* = 5 teeth). Our data were complemented by previously published isotopic data from these sites (**n* *= 88) and from other Brazilian shellmounds (*n* = 51). To quantify the consumption of different food sources, we adapted the Bayesian MixSIAR model to integrate both bone and dentine values without compromising sample independence. Our results indicate that marine fish contributed approximately 30–50% to the diet of individuals from Piaçaguera, and terrestrial animals accounted for 16–45% of the diet of individuals from Moraes. We observed a slight difference in the consumption of marine and terrestrial sources between the burial groups in Piaçaguera and a higher intake of freshwater fish among subadults in Moraes. New radiocarbon dating in Piaçaguera indicates that the two burial groups did not belong to distinct chronological periods. These findings highlight the importance of a constant dialog between archaeological and isotopic data. The adapted MixSIAR model validated and increased the robustness of our results, proving to be effective in delineating dietary patterns in past groups from distinct archaeological contexts.

## Introduction

The hunter-gatherer-fisher groups responsible for building shellmounds along the Atlantic coast have long attracted the attention of South American archaeologists. Known as *sambaquis* in Brazil, these sites date to the Holocene (ca. 8,000–1,500 yBP) and were intentionally constructed by the accumulation of marine mollusk shells mixed with sediments and fish bones [1–3]. These mounds held a significant symbolic value, shaping the identity of a stable and organized society, and providing evidence of human occupation across diverse coastal environments [1,4]. Similar mounds – riverine shellmounds – mainly made of terrestrial mollusk shells are also found inland [5].

Over the past three decades, significant strides have been made in understanding the dietary practices of shellmound-building groups, who exploited both aquatic and terrestrial ecosystems, especially from south-southeastern Brazil. Coastal groups consumed marine and terrestrial resources in varying proportions [6–8], while riverine groups consumed terrestrial and freshwater resources [5,9]. Stable isotopic analysis of carbon (*δ*^13^C) and nitrogen (*δ*^15^N) have confirmed these dietary patterns [10–15].

Despite these advancements, few studies have attempted to quantify the proportion of different food sources in their diets [11,12,15,16], which could contribute to a more robust discussion on dietary variability on regional/temporal scales. Moreover, intra-site variability on diet remains underexplored, as the study on diet of shellmounds from beyond Brazilian southern coast, especially Santa Catarina. Within this context, the Piaçaguera and Moraes sites, both in southeastern Brazil and recently studied for childhood diet [17], offer compelling case studies for investigating intra-site variability.

At Piaçaguera, stratigraphic evidence points to two distinct burial groups [18], which have never been analyzed separately. Investigating these subgroups may reveal potential differences in lifestyle within the same community, as seen in other shellmounds in southern Brazil [19,20]. At Moraes, there is a dietary difference between subadults up to five years old and adults, with a higher intake of freshwater fish by subadults [17]. This age-related dietary variability highlights the complexity of subsistence strategies in this group.

A detailed analysis of intra-site dietary patterns is essential to understanding the social and cultural dynamics of these precolonial groups. Bayesian Mixing Models (BMMs) are particularly useful for estimating the proportional contributions of different food sources to diet. However, they typically operate with one independent isotopic value per individual (e.g., bulk collagen), since their statistical framework depends on the independence of observations. Integrating multiple dependent values (e.g., sequential dentine samples) is challenging, since they are repeated observations from the same individual and do not provide the same informational gain as adding new individuals. Nonetheless, overcoming this limitation could enable more detailed insights into diet over an individual’s life and comparisons across age groups.

This paper aims to investigate dietary variability at the Piaçaguera and Moraes sites by combining *δ*^13^C and *δ*^15^N values from dentine and bone collagen of subadults from 5 years of age, adults, and fauna. For this purpose, new data was produced and integrated with previously published data, and a new adapted version of the Bayesian model MixSIAR was developed to integrate both bone and dentine values, allowing for the estimation of food group proportions across the life course.

By considering the archaeological and funerary contexts when performing isotopic analyses and presenting a new version of MixSIAR, this study contributes to building a more complex understanding of the lifestyle of precolonial South American groups and provides a valuable tool for future research.

## The studied sites

**Fig 1:**
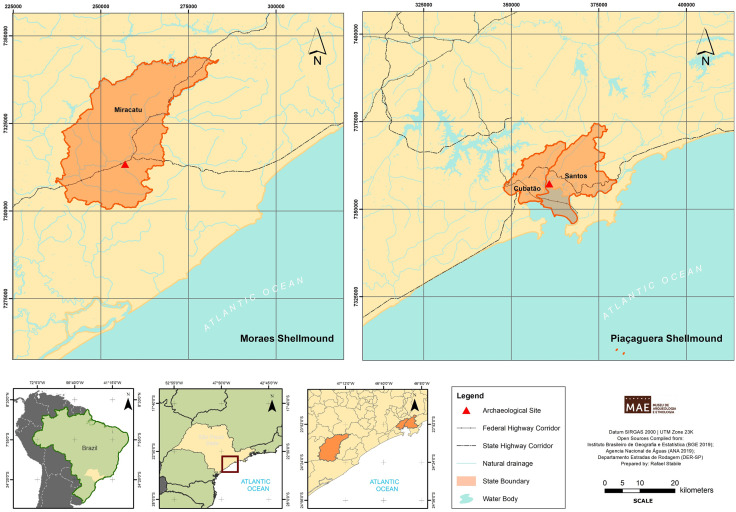
Map of location of Piaçaguera and Moraes shellmounds. Compiled from open data Brazilian sources: Brazilian Institute of Geography and Statistics (IBGE, 2019, https://www.ibge.gov.br/geociencias/organizacao-do-territorio/malhas-territoriais.html), National Water Agency (ANA, 2019, https://metadados.snirh.gov.br/geonetwork/srv/por/catalog.search#/home), and Department of Highways of the State of São Paulo (DER-SP, https://www.der.sp.gov.br/WebSite/Servicos/ConjuntoDados.aspx?tema=Sistema_Rodoviario_Estadual&conjunto=).

Piaçaguera and Moraes are located in São Paulo state, southeastern Brazil (Fig 1). Piaçaguera is on the coast of Santos city (UTM: 23J 0380184/735713), at the bottom of an ancient paleobay near mangroves, sandbanks, the Atlantic Forest, and the Atlantic Ocean, environments rich in biodiversity [6]. Excavations conducted in the 1960s recovered a minimum of 90 human remains, dated between 7,151 and 5,668 yBP [21]. Sex and age analyses were conducted by Fischer [18], based on the criteria proposed by Buikstra and Ubelaker [22] and Schaefer et al. [23].

Previous *δ*^13^C and *δ*^15^N data from adult bone collagen suggest a diet primarily based on both marine and terrestrial protein sources for the Piaçaguera group [24,25], complemented by C_3_ plants, mollusks, and freshwater fish [12]. Isotopic analysis of subadult bone collagen and dentine collagen suggests that weaning likely ended between two and four years of age, and that pregnant women may have consumed slightly more terrestrial resources [17].

**Fig 2:**
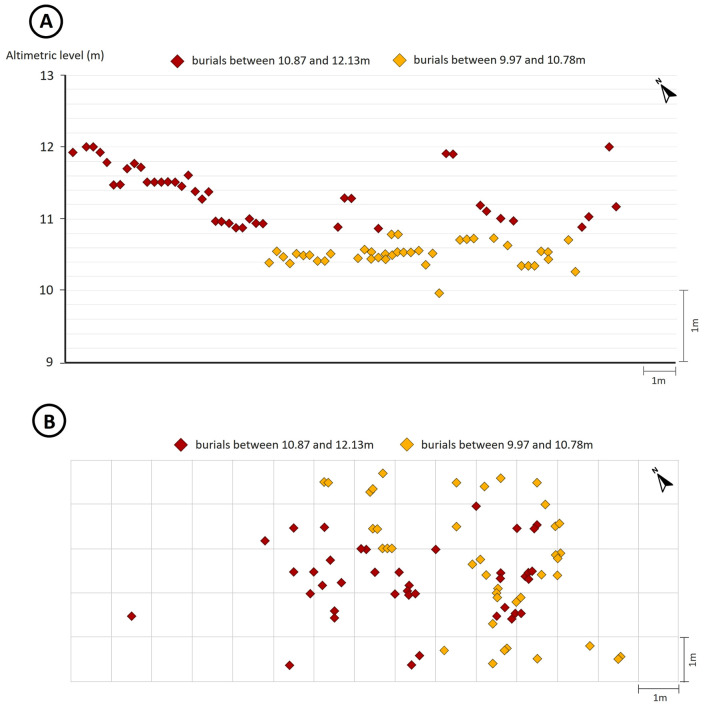
(A) Vertical profile of Piaçaguera with the distribution of human burials (modified from [18,26]). (B) Plan view of Piaçaguera with the distribution of human burials (modified from [18]).

An important feature of Piaçaguera is the presence of two human burial clusters slightly apart from each other both in horizontal space and in stratigraphic sequence: one between 10.87 and 12.13 meters above sea level (here namely Group I), and another between 9.97 and 10.78 meters above sea level (here namely Group II) (Fig 2) [18]. Both clusters include subadults and adults of both sexes.

The Moraes shellmound is located about 25 km inland, in Miracatu city (UTM: 23J 0256908/7313340). Excavations conducted in the 2000s recovered a minimum of 56 individuals, including subadults and adults of both sexes [18], dated between 6,791 and 4,971 yBP [21].

Previous *δ*^13^C and *δ*^15^N analysis of adult bone collagen indicate a diet based on terrestrial animals for the Moraes group, with no significant difference between sexes [14,24,25], complemented by C_3_ plants and mollusks [12]. Isotopic analysis of subadult bone collagen and dentine collagen indicates that weaning likely ended between two and five years of age and that children (and/or pregnant women) may have consumed more freshwater fish [17]. Analyses of starch grains, phytoliths, and lipids indicate an important intake of plant-based resources, including yams (*Dioscorea* sp.) and sweet potatoes (*Ipomoea batatas*), at higher levels than in coastal mound groups in general [27,28].

### The use of *δ*^13^C, *δ*^15^N and mixing models in archaeology

Bulk collagen *δ*^13^C and *δ*^15^N analysis is a widely used method for investigating past diets. The *δ*^13^C is useful for distinguishing between plant groups with different photosynthetic pathways (C_3_, C_4_, and CAM) and between marine and terrestrial diets, while *δ*^15^N reflects trophic levels within food webs, increasing by ~3‰ per level, being useful for identifying dietary preferences across the food web [29,30].

In archaeology, *δ*^13^C and *δ*^15^N for dietary reconstruction often relies on bone and dentine bulk collagen. Bone collagen reflects an average of the protein part of the diet over the last years of life, although this temporal resolution varies depending on the bone element [31] and the individual’s sex [32] and age [33]. In contrast, dentine provides dietary information during specific periods of tooth formation and sequential dentine analysis has emerged as a valuable approach for tracking individual diets across different life stages [17,34–38].

More recently, Stable Isotope Mixing Models (SIMMs) have improved dietary interpretations in past societies [39–41]. Originally developed for ecological research, these models offer a statistical framework to estimate the contribution of different food sources to a consumer’s diet [42]. Among them, BMMs stand out for their capacity to account for the complexities of human subsistence practices, as well as the uncertainties and biases inherent to archaeological samples, particularly those arising from small sample sizes [43].

BMMs operate with independent isotopic inputs, such as bone values, and their application remains limited when applied to dependent data, such as sequential dentine values. Treating dependent values as independent can bias the results and lead to inaccurate estimates, requiring an adaptation to the model that accounts for repeated measures within the same individuals.

One of the most widely used models in archaeology is MixSIAR, an open-source R package [44–49] that, unlike other BMMs, incorporate continuous effects. However, attempts to use this feature in MixSIAR have often failed to produce informative results [39], highlighting its limitations in handling dependent data. Addressing these limitations to incorporate dependent sequential dentine data would allow for the study of diet across an individual’s lifespan, facilitating both inter- and intra-individual comparisons.

## Materials and methods

All necessary permits were obtained for the described study, which complied with all relevant regulations. To date, no indigenous groups in Brazil have recognized the shellmound-builders as their direct ancestors or claimed ethnic and/or cultural connection, and the authorization to analyze the human remains in this study was granted by the National Historical and Artistic Heritage Institute in Brazil (Official letter Nº 733/2021/GAB PRESI/PRESI-IPHAN of February 20, 2021, Process nº 01506.000026/2021–62) and the Museum of Archaeology and Ethnology at the University of São Paulo, which houses these remains.

This study used *δ*^13^C and *δ*^15^N values from new and previously published analysis of human bones (*n* = 67), human teeth (*n* = 28, totaling 190 dentine samples), and faunal bones (n = 92), totaling 349 samples.

Newly analyzed materials include human bones from individuals from six years of age to adulthood (*n* = 19; 5 subadults and 14 adults, including 5 females, 6 males, and 3 adults with indeterminate sex), human teeth with formation periods spanning late childhood (6–12 years) and adolescence (13–18 years) (*n* = 40 dentine slices from 5 teeth; 1 first molar, 1 second molar, 2 third molars and 1 second premolar), and faunal bones from terrestrial (*n* = 18) and aquatic species (*n* = 6) recovered from both sites. Faunal species were identified by one of the authors (JMC) through comparative anatomical analysis, using reference zooarchaeological collections, digital databases, and bibliographic sources [50–53].

Previous analyzed samples include human bones (*n* = 48; 29 subadults and 19 adults, including 9 females, 6 males, and 4 adults with indeterminate sex), human teeth formed during early childhood (birth-6 years) (*n* = 150 dentine slices from 23 teeth; 13 first molars, 3 second molars, 2 third molars, and 5 deciduous teeth), and faunal bones from terrestrial (*n* = 16) and aquatic species (*n* = 1) recovered from both sites [14,17,24].

To complement the fauna dataset, we also included faunal bone values from other Brazilian shellmounds (*n* = 51), selecting those species that are also found at Piaçaguera and Moraes (*n* = 41) or in riverine shellmounds near Moraes (*n* = 10), based on the premise that the riverine sites were part of a culturally connected network [5,9].

At Piaçaguera, individuals were divided between the two burial clusters (Group I and Group II) to investigate potential differences in diet.

The number of samples used in this study is summarized in Table 1, with further details provided in S1 Table in S1 File for human samples and S2 Table in S1 File for faunal samples.

**Table 1:** Summary of newly and previously analyzed human and fauna samples from Piaçaguera, Moraes and other shellmounds used in this study.

Site	Sample	Analyzed in this study	From previous studies	Total
Piaçaguera	Human bones	14	19	33
Piaçaguera	Human teeth	12 dentine samples from 2 teeth (from 2 individuals)	38 dentine samples from 7 teeth (from 6 individuals)	50
Piaçaguera	Fanal bones	10	3	13
Moraes	Human bones	5	29	34
Moraes	Human teeth	28 dentine samples from 3 teeth (from 2 individuals)	112 dentine samples from 16 teeth (from 11 individuals)	140
Moraes	Fanal bones	14	14	28
Other shellmounds	Fanal bones	–	51	51
Total		83	266	349

### Laboratory procedures for isotopic analysis

Bones and teeth were weighed, measured, and photographed using a digital camera (Canon EOS Rebel T5, EF-S 18–55 mm lens). Dental morphological characteristics were described in a brief report, occlusal surfaces were molded with high-precision silicone, and teeth were scanned at CIRIMAT, Université de Toulouse, France. Dental calculus was collected following the protocol described by Wesolowski et al. [54].

Collagen extraction was conducted at the *Laboratoire Méditerranéen de Préhistoire Europe Afrique* (LAMPEA – UMR 7269), Université Aix-Marseille. Bone collagen extraction followed the modified Longin method [55]. Bone chunks (~500 mg) were demineralized in 0.5M HCl at 4°C for four days, treated with 0.125M NaOH, and heated at 75°C in 10^-3^M HCl (pH 3) for 48h. The solution was filtered using a graduated polypropylene tube with a sintered polyethylene filter (Ezee Filter), frozen at −64°C, and freeze-dried for 48h.

For teeth, a 2.5 mm thickness central slice was cut [56,57] and demineralized following Method 2 from Beaumont et al. [34]. Any secondary dentine and the dentine surrounding the pulp chamber and radicular canal were removed. One side of the central slice (mesial or distal) was sampled in sequential horizontal slices with 1 mm thickness, from crown cusp to root apex, following the anatomical location for age-alignment described by Czermak et al. [57]. Each slice was placed in a 1.5 ml microtube with pH 3 solution, centrifuged, frozen at −64°C, and freeze-dried.

Isotopic measurements used 0.5 mg of bone collagen (yield > 8 mg/g) and 0.3–0.5 mg of dentine collagen. Slices with insufficient weight were combined with adjacent slices. Measurements were conducted at IsoAnalytical Laboratory (England) using a Europa Scientific 20−20 IRMS mass spectrometer. Reference materials included IA-R068 (soy protein, *δ*^13^C_V-PDB_ = −25.22‰, *δ*^15^N_AIR_ = 0.99‰) and quality control samples included IA-R068 and IA-R038 (L-alanine, *δ*^13^C_V-PDB_ = −24.99‰, *δ*^15^N_AIR_ = −0.65‰), IA-R069 (tuna protein, *δ*^13^C_V-PDB_ = −18.88‰, *δ*^15^N_AIR_ = 11.60‰), and a mixture of IAEA-C7 (oxalic acid, *δ*^13^C_V-PDB_ = −14.48‰) and IA-R046 (ammonium sulfate, *δ*^15^N_AIR_ = 22.04‰) (S3 Table in S1 File).

Based on Szpak et al. [58] equation, for both series, precision was ± 0.07‰ for *δ*^13^C and ±0.08‰ for *δ*^15^N, accuracy was ± 0.1 for *δ*^13^C and *δ*^15^N, and analytical uncertainty was ± 0.1‰ for *δ*^13^C and *δ*^15^N. Collagen quality followed the criteria stablished by DeNiro [59], Ambrose [60], and Guiry & Szpak [61], namely, carbon and nitrogen content values above 13% and 5%, respectively, and a C/N ratio between 2.9 and 3.6. To verify the absence of correlation between *δ*^13^C or *δ*^15^N values and other variables (carbon content, nitrogen content, C/N ratio, and sample weight), Spearman’s non-parametric correlation test was applied (R Commander package). The non-parametric Mann-Whitney test was used to assess statistically significant differences between group values (sex, age categories, and burial groups).

### Input values in MixSIAR

MixSIAR incorporates isotopic values from both consumers and food sources (divided into food source groups). Consumer values are both *δ*^13^C and *δ*^15^N values measured on human remains from Piaçaguera and Moraes, obtained in this and previous studies (S4 and S5 Tables in S1 File). Adult bone values were used to estimate the overall diet, while subadult bones and dentine slices from 5 years of age onwards were used to estimate diet during childhood. Both subadult bone and dentine samples under 5 years were excluded to avoid potential breastfeeding-related bias in *δ*^15^N, as weaning lasted until 4 years of age in Piaçaguera and 5 years in Moraes [17].

Food source values are both *δ*^13^C and *δ*^15^N values from C_3_ plants and from faunal remains from Piaçaguera and Moraes, obtained in this and previous studies (S6 Table in S1 File), as well as values measured from other Brazilian shellmounds whose species were identified at Piaçaguera and Moraes or at riverine shellmounds near Moraes (S7 Table in S1 File). The faunal values include marine fish (*n* = 31), freshwater fish (*n* = 12), mammals (*n* = 38), and reptiles (*n* = 3). Four main group sources were modeled: marine fish, freshwater fish, terrestrial animals, and C_3_ plants (Table 2). C_4_ plants and mollusks were not included, since there is no significant evidence of C_4_ plants’ consumption by these groups [27] and incorporating mollusks into the Bayesian model presents significant challenges: the organic matter typically constitutes only a minor component of carbonate shells, nitrogen concentrations are typically low, and diagenetic alteration may compromise isotopic integrity [62].

**Table 2:** Categories and descriptions of input values for MixSIAR.

Input Values for MixSIAR		
**Consumers**	**Food Sources**	**Food Source Groups**
*δ*^13^C and *δ*^15^N values from human remains from: • Piaçaguera • Moraes	*δ*^13^C and *δ*^15^N values from C_3_ plants + *δ*^13^C and *δ*^15^N values from faunal remains from: • Piaçaguera • Moraes • other shellmounds	• Marine fish • Freshwater fish • Terrestrial animals • C_3_ plants

C_3_ plants values were obtained from modern fruits (*n* = 30), roots (*n* = 5), and heart of palms (*n* = 13) collected between 2010 and 2013 in southeastern Brazil’s Atlantic Forest national parks (−29.2 ± 3‰ for *δ*^13^C and 1.1 ± 2‰ for *δ*^15^N), whose *δ*^13^C values were corrected for the Suess effect (+2‰) using atmospheric air value from 2010 (−8.4‰) [15,16,63]. Carbon and nitrogen concentration values for each source were obtained from Chinique de Armas et al. [64] and Newsome et al. [40].

Human bone and dentine values were worked together and grouped into age intervals defined by the authors and based on dentine-age growth anatomical areas [17,57,65] (Table 3). These dentine age intervals were used as reference to define broader age categories, starting at 5 years of age. Due to missing values in some age intervals, we merged adjacent age groups and created four broader age-categories used in our analysis: 5–9 years, 9–15 years, 15–18 years, and adults (Table 4; S4 and S5 Tables in S1 File).

**Table 3:** Dentine development approximate age range in years (ys) [57,65].

Tooth	from initial cusp formation to crown completed	from crown completed to bifurcation	from bifurcation to root half	from root half to root completed
1^st^ molar	ca. birth – 3 ys	ca. 3–5 ys	ca. 5–7 ys	ca. 7–9/10 ys
2^nd^ molar	ca. 2–8 ys	ca. 8–9 ys	ca. 9–11 ys	ca. 11–15/16 ys
3^rd^ molar	ca. 9–14 ys	ca. 14–17 ys		ca. 17–19 ys
2^nd^ premolar	ca. 3.5–8.5 ys	ca. 8.5–10.5 ys		ca. 10.5–13.5 ys

**Table 4:** Number of samples per age category for Piaçaguera and Moraes sites.

Age category	N. of samples per site			
	Piaçaguera		Moraes	
	Bone	Dentine slices	Bone	Dentine slices
5-9 years	1	4	2	36
9-15 years	3	14	3	16
15-18 years	–	01	–	12
adults	17	–	16	–

### MixSIAR adaptation to sequential dentine samples

An adaptation of MixSIAR to incorporate correlated values was developed at the Institute of Mathematics and Statistics, University of São Paulo, Brazil. We included in the MixSIAR program *a priori* independent additive random error terms at both individual and sample levels for isotopic measurements, modeling deviations of each individual measurement from the population mean and, when applicable, deviations of repeated measurements from the same individual. This hierarchical approach accommodates both inter- and intra-individual variability, essential for modeling repeated measurements.

Each isotopic value is assumed to follow a conditionally normal distribution, where the average is determined by the weighted average of the tracer values from food sources, adjusted by a trophic enrichment factor. The weights are based on consumption proportions and nutrient concentration variations per food source.

For bone samples, the error from the conditional average includes only the individual-level effect, as there is one measurement per individual. For dentine samples, the error term includes both individual and sample-level effects. The individual-level effect has the same variance as in bone samples and is consistent across multiple samples from the same individual, while the sample-level effect reflects deviations between the sample measurement and the individual’s average values.

We tested the normality assumption for *δ*^13^C and *δ*^15^N values from adult bone samples in each site, since these are the only subgroups with independent and sufficiently large sample size. Lilliefors’ test indicated non-normality for *δ*^13^C values at Moraes (*p* < 0.001). As a robustness check, we repeated the analysis excluding two samples from Moraes with outlying *δ*^13^C values (MO-07 and MO-08) and normality was not rejected without those samples (*p* > 0.2). The comparative analysis found no major changes and we chose to keep the full dataset for the main results. Details of the statistical tests performed and the robustness analysis are in the S4 File.

Parameter estimation was performed using Bayesian inference, with informative prior distributions for all parameters except the main parameter of interest, the consumption proportions, that were chosen as the uniform prior for each subgroup (e.g., age categories) over the 3-simplex, the Dirichlet(1, 1, 1, 1) distribution. This prior is not marginally uniform over each food source, since the consumption proportions must sum to 100%. Each dimension of a Dirichlet(1, 1, 1, 1) distribution follows a marginal Beta(1, 3) distribution, which is asymmetric and decreases over the (0, 1) interval.

Estimation was conducted jointly across all subgroups within the same site, with shared parameters for error variances and mean food source isotope values. Posterior distribution sampling was conducted using the no-U-turn-sampler Markov Chain Monte Carlo algorithm, implemented in Stan software and interfaced through the R programming language. The R and Stan language code is available in the GitHub repository (https://github.com/AFumis/Adapted_MixSIAR_diet_analysis).

Inference on quantities of interest from posterior samples was performed by computing empirical frequencies when possible. For density plots and highest posterior density (HPD) intervals, we used the kernel density estimator (KDE) from the *stats* R package, and incorporated the reflection technique for boundary bias correction [66] when KDE is applied within a limited interval, such as the (0,1) interval for consumption proportions.

### Radiocarbon dating

Since all available radiocarbon dates from Piaçaguera correspond to individuals from Group I, in this study two individuals from Group II were dated at Beta Analytic (USA). Bone samples (~400 mg of ribs) from individuals PI-32 and PI-56 were analyzed using Beta’s in-house NEC accelerator mass spectrometer and four Thermo IRMS units.

Both new and previously published radiocarbon dates from Piaçaguera were calibrated on calib.org platform (8.2 version) considering the corrections for the marine reservoir effect: a ΔR value of −110 ± 25 years, based on ΔR values reported for the region of Santos [67,68], and a mixed marine curve Marine20 and the atmospheric curve SHCal20, adjusted according to the results on marine protein intake identified by the adapted MixSIAR model.

Individual PI-32, aged approximately 15 years old at death, was assigned to the “9–15-year” age category in the MixSIAR model (S4 Table in S1 File). As its radiocarbon date was obtained from rib bone, whose relatively fast turnover rate reflects dietary input from up to the last five years of life [69], we used the MixSIAR result for 9–15-years age category to calibrate this date. For the others dated individuals, all adults, we used the results for adult age categories.

## Results

### *δ*^13^C and *δ*^15^N in bone and dentine collagen

All samples presented in this study meet the quality criteria proposed for archaeological collagen samples (S4-S6 Tables in S1 File). The probability of a correlation between *δ*^13^C or *δ*^15^N values and other variables is low or non-existent at both sites (Spearman’s test: *p* > 0.05; **rho* *< 0.6) (S8 Table in S1 File). The *δ*^13^C and *δ*^15^N values for human bone and dentine and fauna bone are presented in S4-S6 Tables in S1 File and summarized in Table 5.

**Table 5:** Minimum, maximum, average, and standard deviation (SD) δ^13^C and δ^15^N values in human bone and dentine collagens, and fauna bone collagen from Piaçaguera (Groups I and II) and Moraes sites.

Site	Humans/ Fauna	Material	N. of samples	Subgroup	*δ*^13^C_V-PDB_ (‰)				*δ*^15^N_Air_ (‰)			
					min	max	average	SD	min	max	average	SD
PI – Group I	humans	bone	12	adults	−16.7	−14.6	−15.7	0.8	12.1	13.9	13.2	0.5
PI – Group I	humans	bone	8	subadults	−18.4	−15.2	−16.6	1.1	12.4	15.9	13.9	1.1
PI – Group I	humans	dentine	30 slices (5 teeth)	–	−17.6	−14.5	−15.8	0.7	12.6	17	14,0	0.9
PI – Group II	humans	bone	5	adults	−15.9	−12.9	−14.4	1.4	13.0	15.6	14.1	1.0
PI – Group II	humans	bone	8	subadults	−15.9	−13.9	−14.9	0.6	12.8	16.3	15.0	1.2
PI – Group II	humans	dentine	20 slices (4 teeth)	–	−16.2	−14.5	−15.2	0.5	12.4	15.9	14,0	0.8
Piaçaguera	fauna	bone	38	marine fish	−18.4	−8.3	−12.2	2.6	8.6	17.3	12.4	2.1
Piaçaguera	fauna	bone	14	terrestrial animals	−23.5	−13.8	−19.9	2.5	4.9	14.6	8.7	2.9
Moraes	humans	bone	16	adults	−21.3	−19.3	−20.7	0.5	9.6	11.4	10.6	0.5
Moraes	humans	bone	18	subadults	−21.8	−18.8	−20.4	0.8	9.4	14.4	11.9	1.2
Moraes	humans	dentine	140 slices (19 teeth)	–	−23.6	−18.8	−20.6	0.7	9.6	15.6	12.2	1.2
Moraes	fauna	bone	12	freshwater fish	−25.9	−18.5	−23.0	2.1	7.1	12.7	9.8	1.9
Moraes	fauna	bone	27	terrestrial animals	−27.6	−19.6	−22.1	1.6	6.2	12.1	8.4	1.6

**Fig 3:**
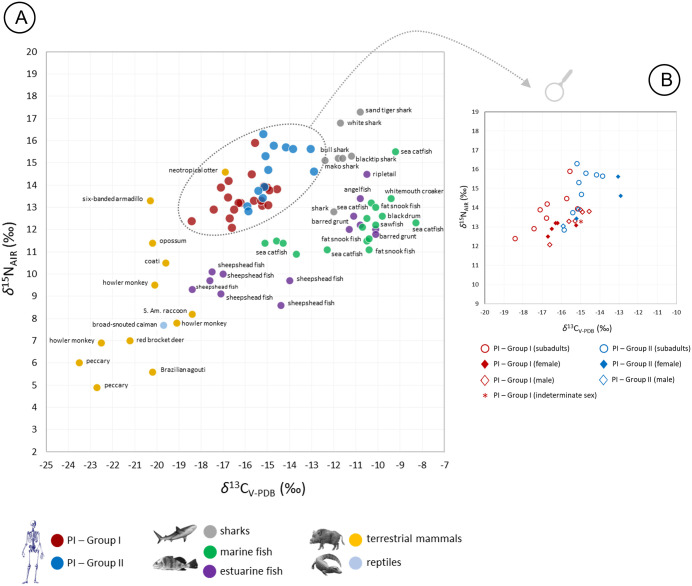
(A) δ13C and δ15N values from bone collagen for humans (Groups I and II) and fauna, Piaçaguera site. The faunal dataset includes values from Piaçaguera as well as other Brazilian shellmounds, and is divided into five broad groups: sharks, estuarine fish, other marine fish, terrestrial mammals, and reptiles. (B) δ^13^C and δ^15^N values from human bone collagen, Piaçaguera site (Groups I and II).

In Piaçaguera, the fauna values dispersion is broad (coefficients of variation of 33% for both *δ*¹³C and *δ*¹⁵N), reflecting the local ecosystem diversity. As shown in Fig 3, trophic level differences are observed: sharks (Elasmobranchii) have the highest *δ*¹⁵N values (12.8‰ to 17.3‰), followed by most marine and estuarine fish (Teostei) (10.9‰ to 15.5‰), with a statistically significant difference between these groups (Mann-Whitney [MW]: *p* = 0.001). Smaller marine fish (e.g., sea catfish [Ariidae]), show lower *δ*¹⁵N (8.6‰ to 10.1‰), significantly different from sharks (MW: *p* = 0.0001) and other marine/estuarine fish (*p* = 0.0004).

Terrestrial fauna has the lowest *δ*¹⁵N values (4.9‰ to 14.6‰), with trophic levels aligning with dietary behavior: carnivorous (e.g., *Euphractus sexcintus* and *Lontra longicaudis*), omnivores (e.g., *Nasua nasua*, *Didelphis aurita*, *Procyon cancrivorus*, and *Tayassu* sp.), and herbivores (e.g., *Alouatta* sp., *Dasyprocta leporina*, and *Mazama* sp.). The otter (*Lontra longicaudis*), whose diet includes fish, sits highest among terrestrial taxa.

Human *δ*¹⁵N values in Piaçaguera fall within the range of most marine and estuarine fish (MW: *p* = 0.05), being lower than shark values (MW: *p* = 0.009) and higher than small marine fish (MW: *p* = 0.007) and terrestrial animals’ values (MW: *p* = 0.0001). Considering an average increase of 3‰ in *δ*¹⁵N and 1‰ in *δ*¹³C per trophic level [29,30], human values are within the range of terrestrial and marine animals, suggesting a diet primarily based on marine/estuarine fish and terrestrial mammals, in line with previous studies [6,24].

Humans from Group I present lower *δ*^13^C and *δ*^15^N values than those from Group II (Fig 3), with statistically significant differences among adults (MW: *δ*^13^C: *p* = 0.001; *δ*^15^N: *p* = 0.004) and subadults for *δ*^13^C (MW: **p* *= 0.004). No significant sex-based differences were found within Group I (MW: *δ*^13^C: *p* = 0.1; *δ*^15^N: *p* = 0.06) or Group II (MW: *δ*^13^C: *p* = 0.1; *δ*^15^N: *p* = 0.2).

**Fig 4:**
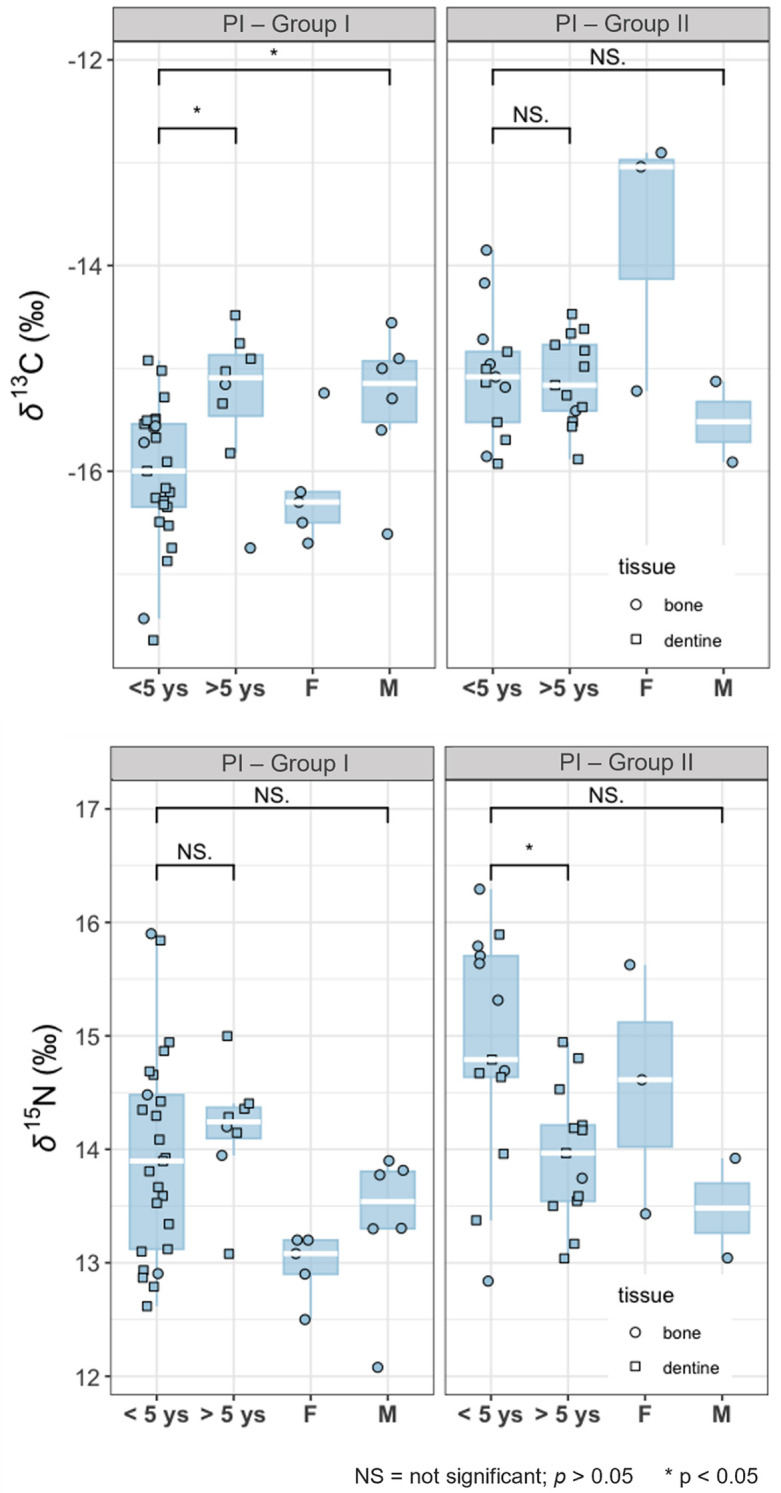
Boxplots of δ^13^C and δ^15^N values of bone and dentine collagen samples from Piaçaguera (Groups I and II). Subadults are divided by age (until and after 5 years) and adults by sex.

Fig 4 shows the distribution of bone values for adults and subadults and dentine values (representing the values from childhood and adolescence), in both Groups I and II. Subadult values were divided by age (under and over 5 years old), since breastfeeding may influence them up to that age.

In both Groups I and II, dentine values closely align with subadult bone values and the *δ*^15^N values until 5 years are higher than those of adults (Fig 4; S1.A – S1.H Figs in S2 File for individual dentine profiles), probably reflecting breastfeeding.

**Fig 5:**
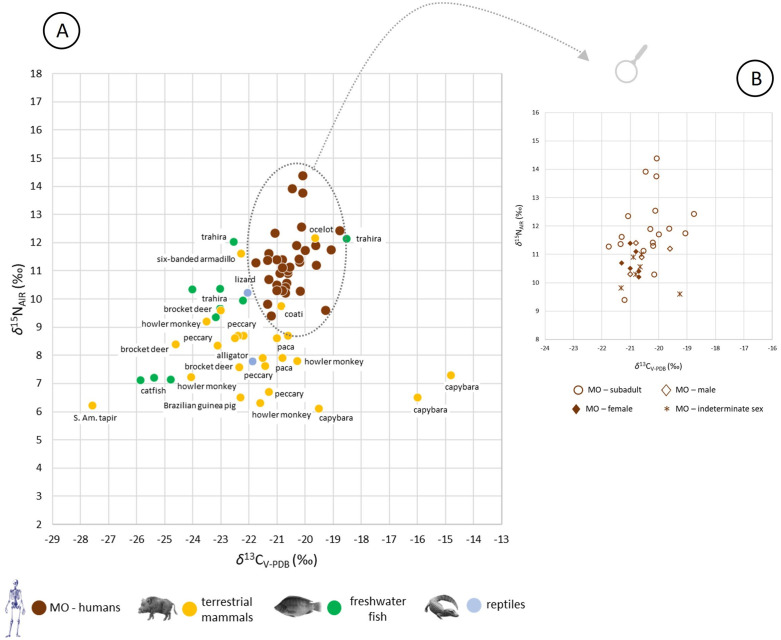
(A) δ13C and δ15N values from bone collagen for humans and fauna, Moraes site. The faunal dataset includes values from Moraes as well as other Brazilian shellmounds, and is divided into three broad groups: terrestrial mammals, freshwater fish, and reptiles. (B) δ^13^C and δ^15^N values from human bone collagen, Moraes site.

**Fig 6:**
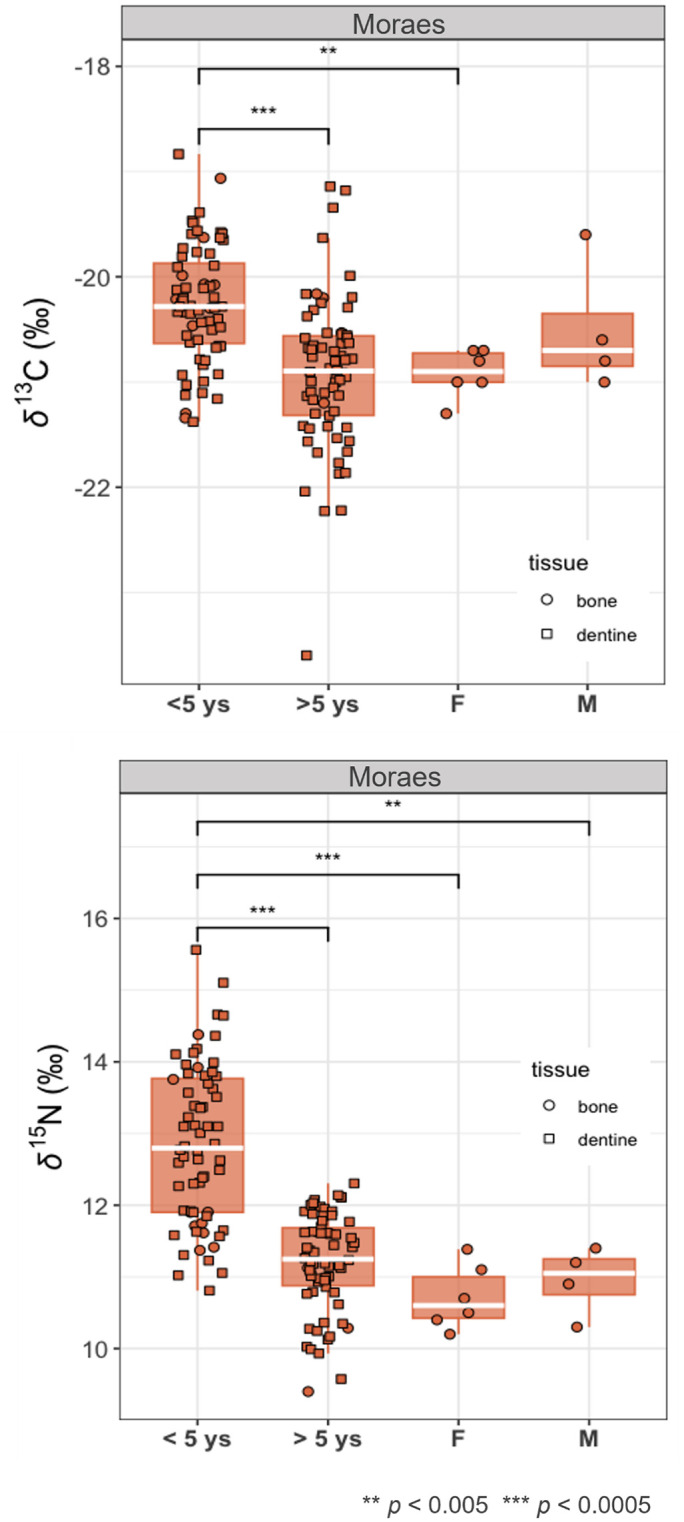
Boxplots of δ^13^C and δ^15^N values of bone and dentine collagen samples from Moraes. Subadults are divided by age (until and after 5 years) and adults by sex.

In Moraes, human bone *δ*^13^C and *δ*^15^N values indicate a predominantly terrestrial-base diet (Fig 5). No statistically significant differences in *δ*^13^C were found between sexes (MW: **p* *= 0.27) or between adults and subadults (MW: **p* *= 0.09). However, a significant difference in *δ*^15^N was observed between adults and subadults (MW: p = 0.0002) (Fig 6).

Dentine values closely align with subadult bone values (Fig 6), with higher variability compared to adult values (Fig 6; S2.A – S2.M Figs in S3 File for individual dentine profiles). *δ*^15^N values until 5 years are significantly higher than those of adults, probably reflecting breastfeeding potentially combined with a higher freshwater fish intake by children and/or lactating women (17). Even in the > 5 years old group, some subadults show higher *δ*^15^N values than the adults (MW: *p* = 0.0001) (S2.A – S2.M Figs in S3 File).

### MixSIAR and diet modeling

For Piaçaguera, two models were developed: Model 1 grouped all individuals, while Model 2 separated them into Group I and Group II. The 15–18 years age category was excluded from the analysis due to insufficient sample size (Table 4).

**Fig 7:**
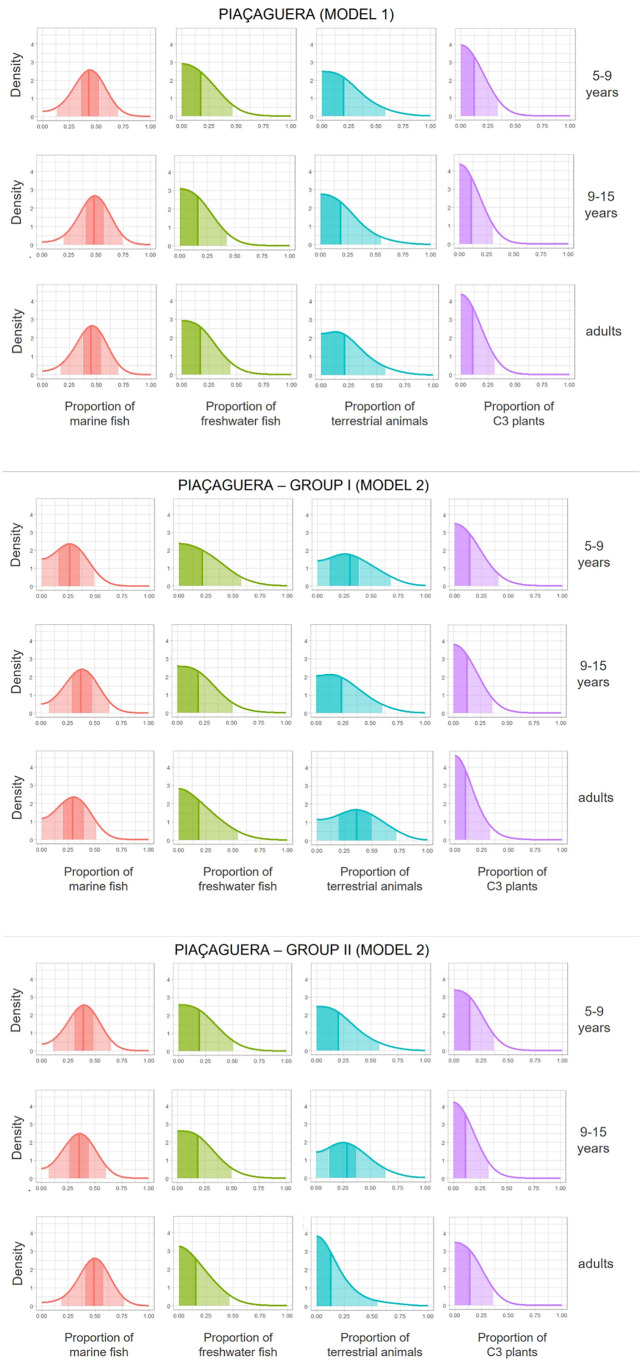
Posterior density estimates of the distributions of food source group consumption for age categories for Piaçaguera individuals, with 50% and 95% probability HPD intervals and the median.

In Model 1, marine fish is the primary dietary source across all age groups, whereas C_3_ plant contributes the least (Fig 7). As shown in S9 Table in S1 File, freshwater fish and terrestrial animals have modes at or near zero across all age categories, but with higher dispersion than C_3_ plants. A slight variation is observed in the 9–15 years category, where HPD interval limits and quartiles for marine fish are approximately 4 percentage points higher, and those for terrestrial animals about 3 percentage points lower than in other age categories.

Some individuals could not be assigned to either Group I or II due to the lack of burial stratigraphy data, increasing the sample size in Model 1. Nonetheless, this did not affect the results, with dietary proportions remaining consistent.

In Model 2, Group I and II show the posterior distributions that indicate different primary food sources. Group I shows terrestrial animals as the primary source for subadults and marine fish for adults, while C_3_ plants as the lowest source for all age categories (Fig 7). Group II shows marine fish as the primary source and C_3_ plants as the lowest for all age groups. When comparing the posterior distribution between Groups I and II, Group I has higher estimates for terrestrial animal intake across all age categories, indicating higher consumption of terrestrial animals in Group I than in Group II.

Group I shows lower values for marine fish consumption than Group II for adults (Group I quartiles: 0.185, 0.292, 0.387; Group II quartiles: 0.395, 0.488, 0.569) and for 5–9 years category (Group I quartiles: 0.161, 0.262, 0.360; Group II quartiles: 0.295, 0.388, 0.474). For 9–15 years category, both Group I and II show similar values for marine fish (Group I quartiles: 0.272, 0.373, 0.466; Group II quartiles: 0.256, 0.352, 0.441) (S10 and S11 Tables in S1 File). Considering these posterior distributions and using the highest third quartile between age groups as a benchmark, we can estimate that the proportion of marine fish may reach up to 39% for adults and 46% for subadults in Group I, and up to 57% for adults and 47% for subadults in Group II.

The values for terrestrial animal consumption are higher in Group I than Group II for adults (Group I quartiles:0.113, 0.212, 0.340; Group II quartiles: 0.056, 0.127, 0.243), for 5–9 years category (Group I quartiles: 0.170, 0.304, 0.459; Group II quartiles: 0.101, 0.203, 0.337), and for 9–15 years category (Group I quartiles: 0.210, 0.359, 0.513; Group II quartiles: 0.165, 0.285, 0.420) (S10 and S11 Tables in S1 File). It can be estimated that the proportion of terrestrial animals in the diet may reach up to 34% for adults and 51% for subadults in Group I, and up to 24% for adults and 42% for subadults in Group II.

When comparing subadults between Model 1 and 2, for both the 5–9 years and 9–15 years categories, the overall sample (Model 1) shows higher estimates for marine fish consumption and lower estimates for terrestrial animal consumption than Groups I and II (Model 2) (Fig 7; S9-S11 Tables in S1 File).

**Fig 8:**
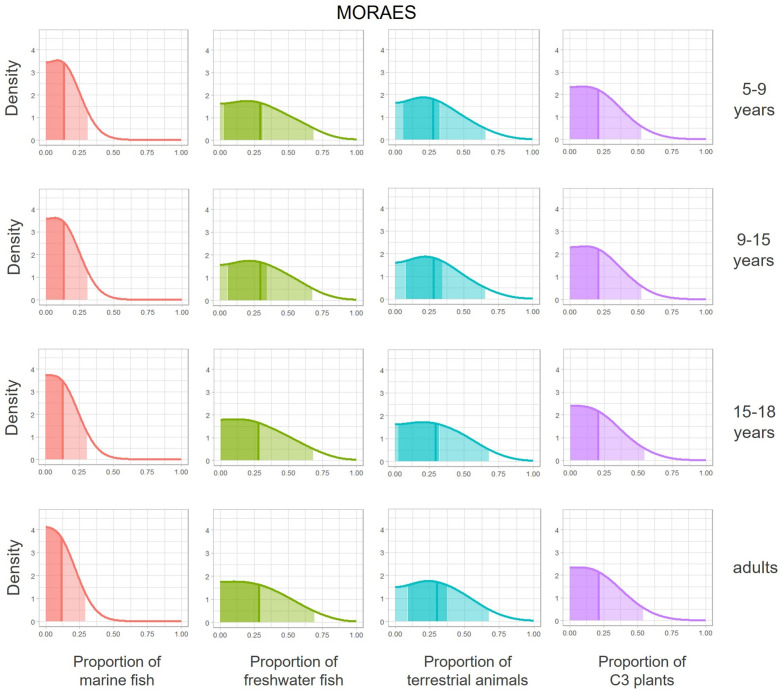
Posterior density estimates of the distributions of food source group consumption for age categories for Moraes individuals, with 50% and 95% probability HPD intervals and the median.

In Moraes, terrestrial animals are the main food source across all age categories, followed by freshwater fish and C_3_ plants (Fig 8). Terrestrial animal intake is highest among adults, particularly when compared to the 5–9 years and 9–15 years categories, with the highest lower and upper limits 50% HPD interval (0.091, 0.377) and quartiles (0.163,0.301,0.459), matching the 15–18 years category in the third quartile (0.459) (S12 Table in S1 File). The posterior distribution shifts rightward with age, indicating increased terrestrial animal source proportion (Fig 8). It can be estimated that the proportion of terrestrial animals in the diet of Moraes may reach up to 46% for adults and 43% for subadults up to 15 years old.

Conversely, freshwater fish consumption has the highest lower and upper limits of the 50% HPD interval for the 5–9 years and 9–15 years categories (0.024, 0.314 for 5–9 years and 0.056, 0.345 for 9–15 years), compared to the 15–18 years (0, 0.279) and adult categories (0, 0.287) (S12 Table in S1 File). It can be estimated that the proportion of freshwater fish may reach up to 45% for adults and 46% for subadults up to 15 years old.

C_3_ plant intake is consistently lower than that of terrestrial and freshwater sources across all age categories, but higher than in Piaçaguera. For instance, adults in Moraes show higher quartiles (0.108, 0.213, 0.341) than Piaçaguera’s Model 1 (0.052, 0.108, 0.182) and Model 2 (Group I: 0.044, 0.098, 0.175; Group II: 0.071, 0.142, 0.226) (S9-S12 Tables in S1 File). The estimated proportion of C_3_ plant in the diet may reach up to 20% for Piaçaguera (Groups I and II) and 30% in Moraes.

Interestingly, marine fish consumption is minimal across all categories in Moraes, with 50% HPD limits typically below 13% and unimodal distributions centered near zero (S12 Table in S1 File), suggesting minimal or no consumption of marine fish.

### Radiocarbon dates

The adapted MixSIAR model estimated marine protein intake of up to 40% for adults from Group I, 60% for adults from Group II, and 45% for the 9–15 years age group from Group II (S10 and S11 Tables in S1 File). These values were applied during calibration using a mixed Marine20 and atmospheric SHCal20 curve.

Table 6 presents all the radiocarbon dates for Piaçaguera (divided in Group I and II) and Moraes. The new dates published here regarding Group II suggest a possible contemporaneity between individuals from Group I and Group II.

**Table 6:** Radiocarbon dates for human skeletal remains from Piaçaguera and Moraes shellmounds.

Site	Subgroup	Burial	Sample	Age (uncal. BP)	Age (cal. BP, 95.4%)	Reference
Piaçaguera	Group I	1A	Beta− 599619	5290 ± 30	5941 − 5703	This study (after [12,21])
Piaçaguera	Group I	5	AA- 109292	6342 ± 34	7163 − 6877	This study (after [12,21,70])
Piaçaguera	Group I	15	AA- 109293	5437 ± 31	6124 − 5904	This study (after [12,21,70])
Piaçaguera	Group II	32	Beta-734866	5500 ± 30	6191 − 5927	this study
Piaçaguera	Group II	56	Beta-734867	5670 ± 30	6288 − 6010	this study
Moraes	Moraes	05	KIA- 15562	4985 ± 35	5851 − 5590	[12,21,71]
Moraes	Moraes	05	MAMS-34575	5092 ± 23	5906 − 5665	[12,72]
Moraes	Moraes	13	KIA- 15561	5895 ± 45	6792 − 6502	[12,21,71]
Moraes	Moraes	25	KIA- 20844	4511 ± 32	5298 − 4971	[12,21,71]
Moraes	Moraes	37	KIA- 20843	5420 ± 30	6286 − 6007	[12,21,71]

## Discussion

### The diet in Piaçaguera shellmound group

Isotopic values from adult bone collagen at Piaçaguera indicate a diet based on the consumption of marine and terrestrial animals, aligning with previous isotopic and zooarchaeological studies [6,24]. Similar values are found in other archaeological groups with mixed marine/terrestrial diets, such as those from Argentine coast of Patagonia [73–75].

However, Piaçaguera *δ*^13^C and *δ*^15^N values are lower than those from other Brazilian coastal shellmounds where marine/estuarine fish intake was more significant (e.g., Morro do Ouro, Rio Comprido, and Enseada I sites in northern Santa Catarina [10,15,16]), and even lower than in groups with predominantly marine diets (e.g., Sambaqui do Moa in Rio de Janeiro state and Jabuticabeira II, Capivari I, Cabeçuda, Armação do Sul, and Galheta IV sites in central and southern Santa Catarina [7,11–13,24,76,77]. This reinforces the significant contribution of terrestrial resources to the diet in Piaçaguera.

Zooarchaeological studies suggest a diet based on small marine and estuarine fish and local terrestrial fauna [6]. Our results confirm this, pointing to low-trophic-level fish from shallow or estuarine waters (e.g., catfish [*Genidens barbus*], black drum [*Pogonias cromis*], snook [*Centropomus* sp.], and whitemouth croaker [*Micropogonias furnieri*]) as major protein sources. Terrestrial sources included small mammals from the Atlantic Forest (e.g., coatis [*Nasua nasua*], raccoons [*Procyon cancrivorus*], guinea pigs [*Cavia aperea*], and howler monkeys [*Alouatta* sp.]) (Fig 3). This local-resource pattern is consistent with other isotopic studies from Brazilian shell mounds [10,13,15,16], and Piaçaguera is no exception.

Some species found at the site, such as alligators, sharks, and open-sea fish (e.g., tripletail [*Lobotes surinamensis*] and angelfish [*Pomacanthus paru*]), may have been consumed opportunistically or during special occasions. Borges [6] suggests that higher sea levels at the time may have brought open-sea animals closer to the settlement, increasing their availability. Klökler [78] also notes the frequent use of shark teeth as tools and ornaments in burials, suggesting that their occasional consumption may have been intentional.

The adapted MixSIAR model confirms marine fish as the main protein source, contributing around 30–50% of the diet. Terrestrial animals and freshwater fish accounted for 10–30%, while C_3_ plants contributed around 10%. Since bulk collagen reflects mostly the protein component of the diet, the contribution of plant-based foods may be underestimated. Protein content in collagen is mainly assessed through nitrogen, which plants supply at relatively low levels, particularly due to the limited digestibility of a substantial portion of the nitrogen sources they contain [79,80].

Due to this limitation, it is important to consider other methods for inferring plant consumption in a population, such as the identification of plant microremains in dental calculus or, indirectly, through patterns of dental wear and the presence of caries. Although carbohydrate intake was not directly studied in Piaçaguera, nor was dental wear analyzed for dietary purposes, dental caries are rare in this group [81]. Caries etiology is complex and multifactorial, but frequent intake of fermentable carbohydrates is a key contributor [82–84]. Their low prevalence in the group suggests that plant-based foods were probably less important than in other shell mound groups from southern Brazilian coast [84,85].

When grouped by burial location, Group II individuals show slightly higher *δ*^13^C and *δ*^15^N values than those in Group I (Table 6; Figs 3 and 4), with a statistically significant difference for *δ*^13^C (MW: *p* = 0.001). Both groups had mixed marine/terrestrial diets, but Group I appears to have consumed more terrestrial resources. This is supported by the adapted MixSIAR model, which shows higher density values for terrestrial animal intake in Group I (Fig 7). Two hypotheses may explain these dietary differences:

Scenario 1 – Environmental change: The Holocene sea-level regression along the Brazilian Atlantic coast [86,87] may have increased terrestrial fauna availability, leading to higher consumption of this resource by Group I. A similar scenario has been proposed for Sambaqui do Moa, Rio de Janeiro [11]. If this were the case, Group I would represent a more recent occupation of the site. However, new radiocarbon dates presented here for two individuals from Group II, ranging between 6,288 and 5,927 yBP, show that Group I and II were contemporaneous, suggesting that the slight change in diet was more probably due to cultural factors than environmental changes.

Scenario 2 – Cultural factors: Affinity groups based on varying degrees of kinship, neighborhood associations, or other social principles has been proposed for the shellmound Jabuticabeira II, located on Brazil’s southern coast [88,89]. Social structures potentially similar to those proposed for Jabuticabeira II, such as affinity groups, may also have existed at Piaçaguera and could explain both the distinct burial areas and differences in diet, with each group having differential access to terrestrial or marine resources.

Among Group I individuals, females (*n* = 5) showed slightly lower isotopic values than males (*n* = 6) (Figs 3 and 4), although this difference was not statistically significant (MW: *δ*^13^C: *p* = 0.1; *δ*^15^N: *p* = 0.06). This may reflect a slightly higher intake of terrestrial resources by females, possibly due to dietary restrictions/preferences or different access to food resources. While osteological stress markers associated with physical activities in Piaçaguera found no significant sex-based differences [90,91], different tasks – that could involve similar physical efforts – may have led to differential food access, for example, males responsible for fishing and females for hunting. Furthermore, the presence of individuals from outside the group cannot be excluded.

In both Groups I and II, subadult isotopic values are generally similar to those of adults, except for *δ*^15^N in children under five, which may be influenced by breastfeeding [17]. From age six onward, *δ*^15^N values align closely with those of adults (S1.A-S1.H Figs in S2 File), indicating that children and adolescents also had a mixed marine/terrestrial diet. The adapted MixSIAR model supports these findings, showing similar proportions of resource consumption between subadults and adults in both groups (Fig 7).

Notably, the higher intake of terrestrial resources observed in Group I adults was also observed in Group I subadults from age six onward (Fig 7; S10 Table in S1 File). Within scenario 2 hypothesis, which proposes that social structures like affinity groups influenced diet, these groups would have been established early in life, meaning that social organization and its impact on diet were already present from childhood.

Minor individual variations in isotopic values during childhood and adolescence in both Groups I and II (S1.A-S1.H Figs in S2 File) may reflect slight variabilities in the intake frequencies of terrestrial/marine resources and/or punctual periods of metabolic alterations, which could influence *δ*^13^C and *δ*^15^N values [92,93].

Human skeletal series dating over millennia are often treated as homogeneous groups, as was the case of Piaçaguera. However, such series are not representative of the entire population [94], and radiocarbon dating can help contextualize the individuals temporally. While financial constraints may restrict access to such analysis, archaeological information (e.g., spatial distribution of burials) is also essential to contextualize the individuals and reduce errors associated with homogenizing entire series. In this study, dividing human burials into clusters based on their stratigraphy revealed significant isotopic variations in diet, highlighting the importance of a constant dialogue between archaeological and isotopic data.

### The diet in Moraes shellmound group

Isotopic values from adult bone collagen at Moraes suggest a predominantly terrestrial diet, in line with previous isotopic and zooarchaeological studies [14,24]. Unlike Piaçaguera, no distinct burial groups were identified at Moraes [95], and all radiocarbon-dated individuals show similar isotopic values (Table 5 and S4 Table in S1 File), suggesting certain stability in diet within the group.

The *δ*^13^C and *δ*^15^N values from Moraes are similar to other archaeological groups that relied primarily on terrestrial animals (e.g., Argentine Patagonia [96]), but higher than those of groups relying mainly on terrestrial herbivores (e.g., Archaic-period hunter-gatherers from Chile [97]), C_3_ plants (e.g., groups from Alto Jararaca II and Gruta do Rio dos Altos sites, southern Brazil [98]), and C_3_ plant horticulturalists (e.g., CAI period in Chile [97]).

Considering (a) the estimated trophic enrichment in ¹⁵N and ¹³C between consumer and food source values [29,30], (b) the zooarchaeological record from Moraes [14], and (c) the isotopic values of humans and fauna from the site (Fig 5), the main protein sources for the Moraes group were most likely small- and medium-sized terrestrial mammals from the Atlantic Forest. These include lowland paca (*Cuniculus paca*), South American coati (*Nasua nasua*), peccary (*Tayassu* sp.), and howler monkey (*Alouatta* sp.). These findings align with evidence of low long-range mobility for this group [99] and with zooarchaeological data from nearby riverine shell mounds [9].

Some species, such as tapir (*Tapirus terrestres*), alligator (*Caiman latirostris*), and ocelot (*Leopardus* sp.), may have been consumed opportunistically or in specific cultural contexts. Freshwater fish also contributed to the diet but likely played a second role compared to terrestrial mammals.

The adapted MixSIAR model support these findings, indicating that terrestrial animals contributed approximately 16–45% of total protein intake, freshwater fish accounted for 14–45%, and C_3_ plants contributed 10–34%. The C_3_-plant intake is also supported by the high prevalence of dental caries in the group [14] from results provided by one of the authors [VW]), and by the presence of starch/phytoliths in dental calculus [99], associated with the consumption of yams (*Dioscorea* sp.) and sweet potatoes (*Ipomoea batatas*) [27].

Although the adapted MixSIAR model points to a minimal marine fish intake in Moraes, this result requires careful interpretation. Evidence of contact between Moraes and coastal groups is proved by the presence of marine mollusk shells and perforated shark teeth (*Carcharinus plumbeus*) at the site associated with some human burials [14]. However, this interaction does not appear to have been extensive enough to include, for example, intergroup marriage [21,100].

Marine species could have been brought to Moraes for consumption, or individuals from the group may have traveled to the coast and brought to the site marine materials as symbolic artifacts, but their presence in the dietary modeling does not necessarily confirm their regular consumption in Moraes. *δ*^15^N values from some estuarine/marine fish (e.g., *Archosargus probatocephalus*) can overlap with those of large carnivores’ freshwater fish, such as freshwater catfish (Actinopterygii) and trahira (*Hoplias* spp.) (Fig 5; S6 and S7 Tables in S1 File), potentially leading to ambiguous results. Therefore, BMM results in archaeological studies require cautious interpretation, relying on contextual information about the analyzed population.

Subadult isotopic values also indicate a terrestrial-based diet. However, while *δ*^13^C values remain similar between adults and subadults of all ages, *δ*^15^N shows a different and interesting pattern. For children under five years old, *δ*^15^N values exceed the typical 3‰ increase with breastfeeding (Fig 6; S2.A-S2.M Figs in S3 File). As proposed by DiGiusto et al. [17], this elevation could also result from a higher intake of freshwater fish (which present higher *δ*^15^N values than terrestrial animals) by young children and/or nursing women, not only breastfeeding. The Moraes group exhibits a high neonatal mortality rate, and freshwater fish could have served as a source of nutritional support during childhood [17].

From the age of five onwards, *δ*^15^N values remain higher than those of adults (Fig 6; S2.A-S2.M Figs in S2 File), as confirmed by the adapted MixSIAR model, that indicates higher proportions of freshwater fish in the diet of subadults than in adults. As subadults’ age increases, the proportions of freshwater fish decrease and terrestrial animals increase (Fig 8). These findings highlight a clear dietary difference between adults and subadults at Moraes and reflect a community strategy aimed at ensuring the survival of subadults.

No significant differences in isotopic values were found between females and males in the group, indicating no dietary differences between sexes. However, osteological stress marker analysis suggests possible differences in activity patterns in Moraes. Males showed higher osteoarthritis frequencies in the upper limbs, indicating more intense and/or frequent use of the upper limbs by them, and auditory exostosis (bony growths of the external auditory meatus, frequently associated with aquatic activities involving diving in cold water) was observed exclusively in males [99].

Considering the terrestrial-based diet of the group and a possible higher freshwater fish intake by subadults and/or nursing women, these activity markers may be related to male-dominated fishing activities, possibly involving diving and sailing. This suggests a male food provisioning system for fish resources for subadults and/or nursing women. Male provisioning for maternal and childhood diets has been proposed for some South American hunter-gatherer groups [101] and is documented in ethnographic studies of the Hazda and Hiwi ethnic groups in Africa [102,103]. A similar dynamic may have been present in the Moraes group.

### An evaluation of MixSIAR adaptation for use bone and dentine collagen isotopic values

The introduction of additive error terms in the model’s likelihood function allows for the inclusion of repeated samples from the same individual without violating the independence assumptions between samples. This approach mitigates information loss that would result from using intra-individual averages instead of the full set of measurements, and enables the simultaneous analysis of bone samples (one observation per individual) and dentine samples (multiple observations per individual).

Similar to the original MixSIAR, this adapted model may become overly dependent on prior choice when the number of food sources exceeds the number of isotopic measurements by more than one. This was the case in our study. Stock et al. [104] discuss best practices in such situation, recommending that all food sources be kept in the model and aggregating posterior consumption proportions if needed.

This dependency on the prior is a limitation that raises challenges for consistency in estimates when subdividing the sample, since the number of measurements per group is reduced – another situation where the prior tends to dominate. This is illustrated by the slightly higher marine fish consumption for subadults in Piaçaguera in Model 1 (without divide the group in Group I and II) in comparison with Model 2 (where the groups divided in Group I and II). The higher total sample size in Model 1 allows to obtain a posterior distribution more distinct from the prior than either of the subpopulations separately in Model 2.

Another constraint concerns the identification of food sources with overlapping isotopic values, such as small marine or estuarine fish and large freshwater fish, as observed in the Moraes model. However, this limitation can be mitigated by archaeological contextual information, which aids in interpreting BMM results.

Despite these limitations, BMMs remain a strong alternative to enhance dietary interpretations based on isotopic analysis.

The model presented here was capable to produce distinct posterior distributions for the analyzed human groups from the same prior, despite potentially small sample sizes in certain categories. Its agreement with qualitative analysis of archeological remains reinforces its utility and serves as a useful tool to measure the consumption of different food sources through the life of individuals.

## Conclusion

In Piaçaguera, the data revealed a mixed marine and terrestrial diet, with notable differences in diet between the individuals from Groups I and II. Based on the new radiocarbon dates, the higher intake of terrestrial resources by Group I may be attributed to social structures within the community, that could have influenced resource consumption since childhood. This highlights the importance of integrating isotopic and archaeological data to achieve a comprehensive understanding of past dietary practices.

In Moraes, the group had a predominantly terrestrial diet with significant age-related differences. Subadults showed a higher consumption of freshwater fish, reflecting a community strategy aimed at ensuring the nutritional necessities of subadults and nursing women, with a possible male provisioning system for fish resources.

The adapted MixSIAR model, as well as the original MixSIAR, has inherent complexities. Despite this and the limitations of the samples themselves (such as the total number of samples available and the lack of samples at shorter age intervals), this adaptation has proven effective in delineating dietary patterns within archaeological groups, by increasing the number of measurements in the model, adding bone and dentin isotopic values together, and being able to present the sources of food consumed by individuals throughout their lives at different ages. Its ability to incorporate both bone and dentine collagen values at the same time without compromising the independence of the samples increases its robustness. While dependence on prior distributions remains a challenge, the model’s ability to align with archaeological evidence and identify dietary patterns in distinct archaeological contexts validates its utility.

Future research into other isotopic elements has the potential to further illuminate social dynamics within these groups, elucidating the factors influencing their dietary practices.

## Supporting information

S1 File S1-S12 Tables. Materials, isotopic results, and MixSIAR results.
(XLSX)

S2 File S1.A-S1.H Figs : Individual dentine δ^13^C and δ^15^N isotope ratio profiles for Piaçaguera.
(PDF)

S3 File S2.A-S2.M Figs : Individual dentine δ^13^C and δ^15^N isotope ratio profiles for Moraes.
(PDF)

S4 File Normality test. (PDF)
