# Supplementary material for: Dietary variability in Middle Holocene South American shellmounds: Insights from isotopic analysis and an adapted Bayesian MixSIAR model
Source: PLoS One. 2025 Dec 3;20(12):e0335680. doi: 10.1371/journal.pone.0335680 (PMC12674525; doi:10.1371/journal.pone.0335680)

S1 (A-H) Fig: Individual dentine $\delta^{13}C$ (in blue) and $\delta^{15}N$ (in red) isotope ratio profiles for the Piaçaguera series.

Fig S1.A: Individual PI-04.

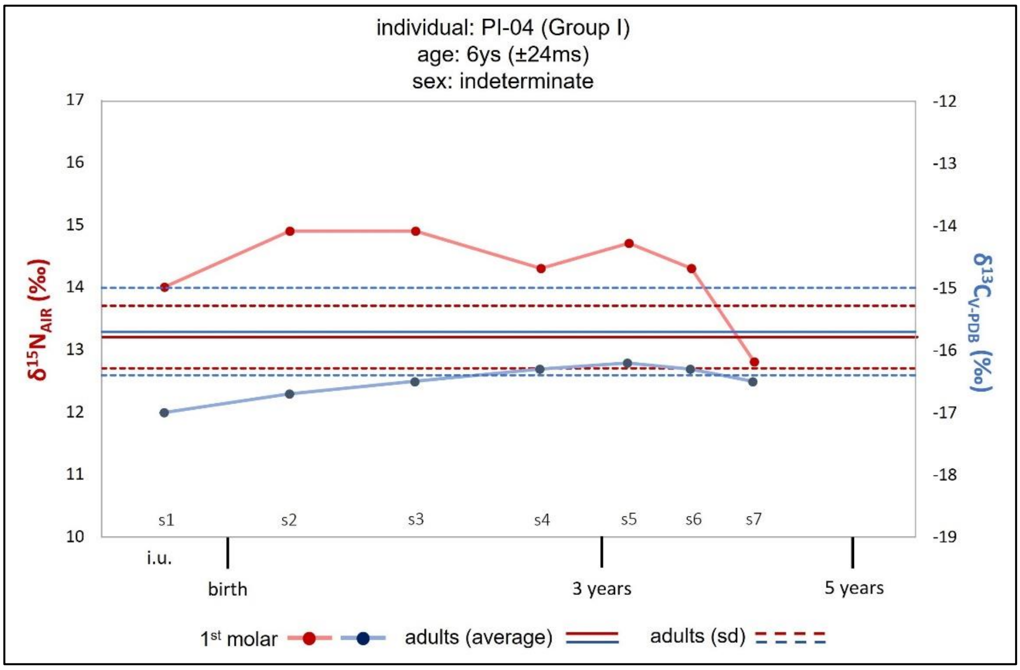

Fig S1.B: Individual PI-05.

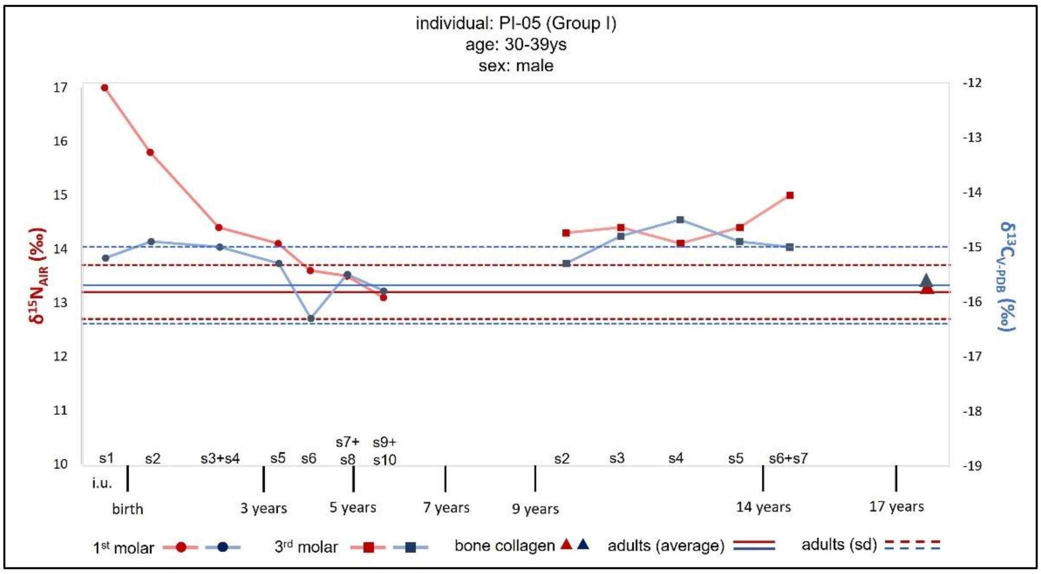

Fig S1.C: Individual PI-15.

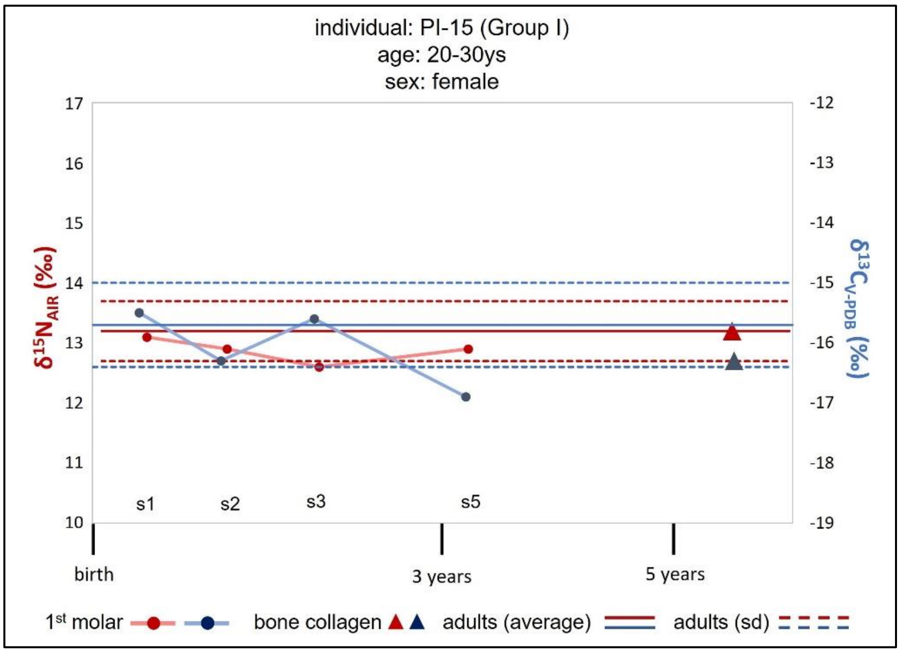

Fig S1.D: Individual PI-19.

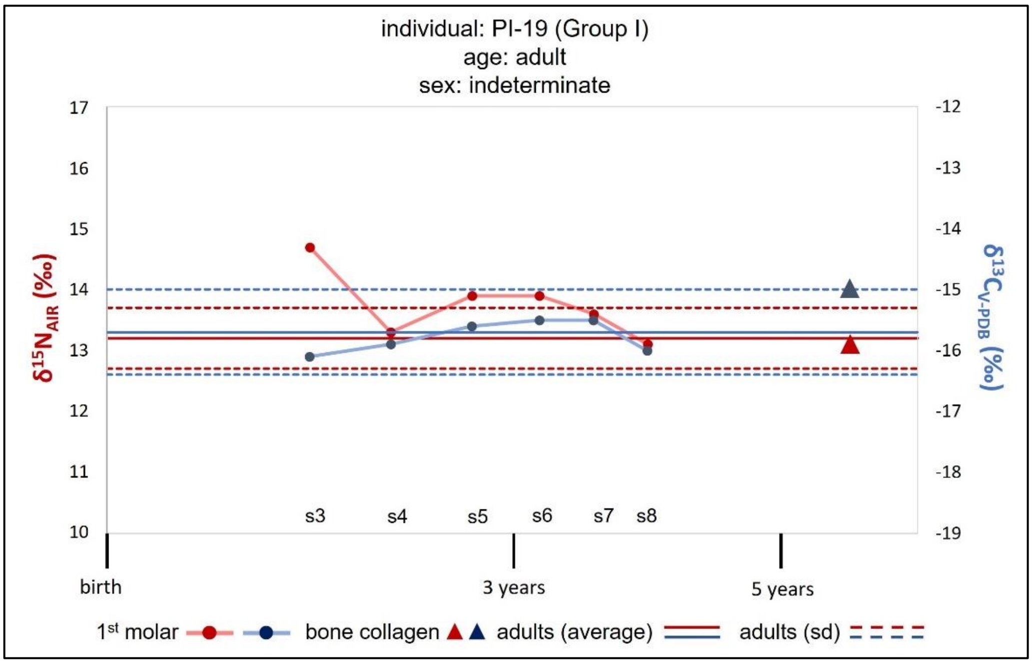

Fig S1.E: Individual PI-32.

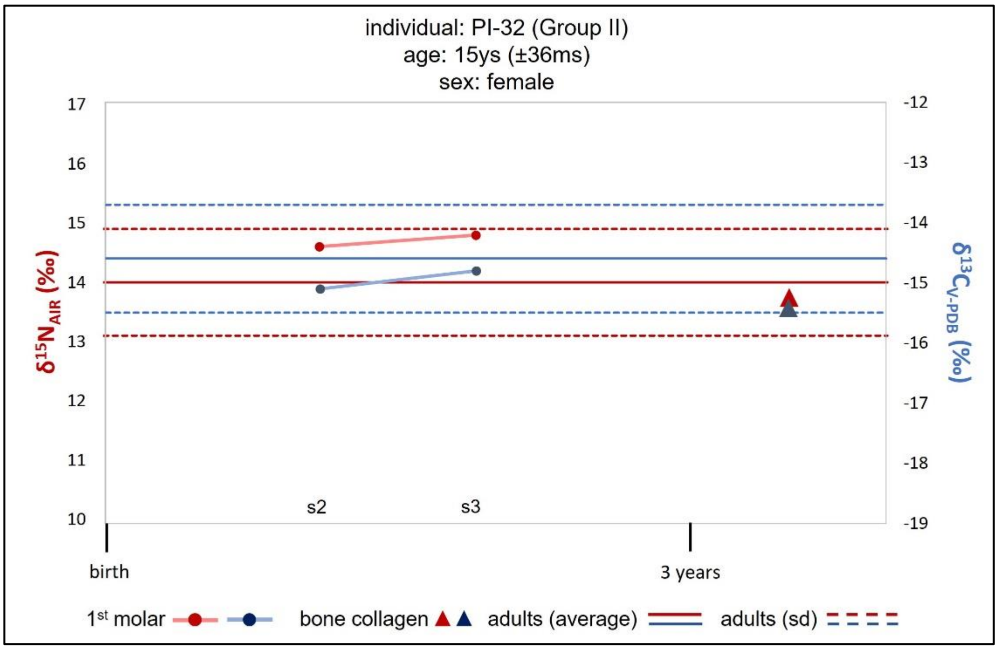

Fig S1.F: Individual PI-44.

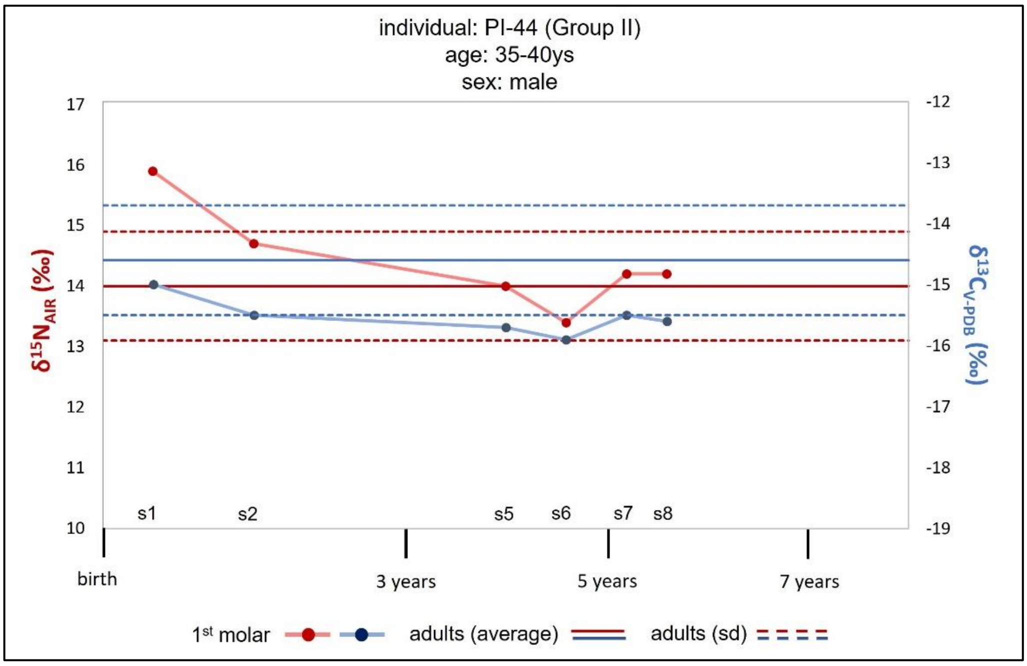

Fig S1.G: Individual PI-52.

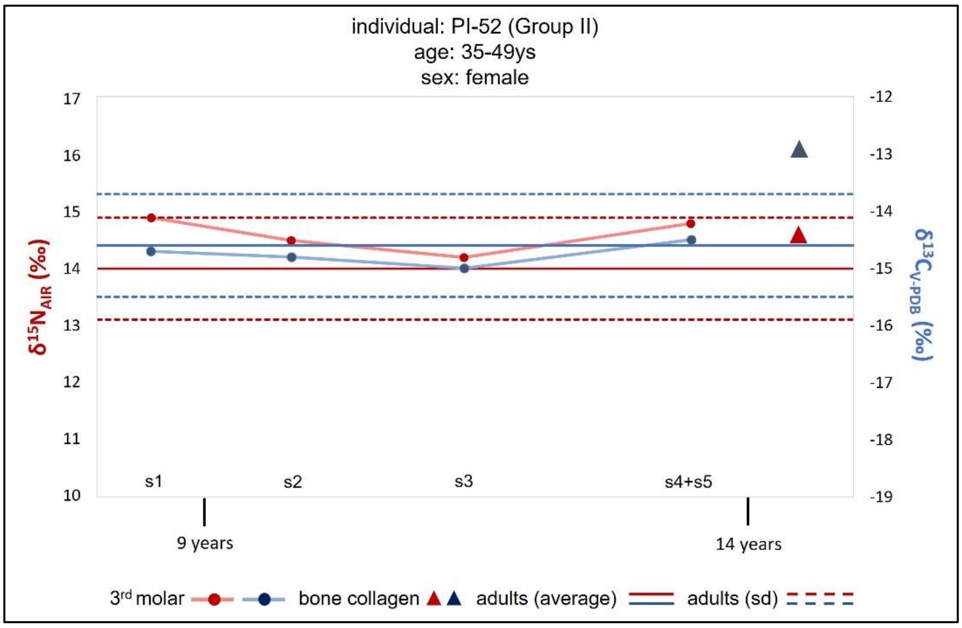

Fig S1.H: Individual PI-53.

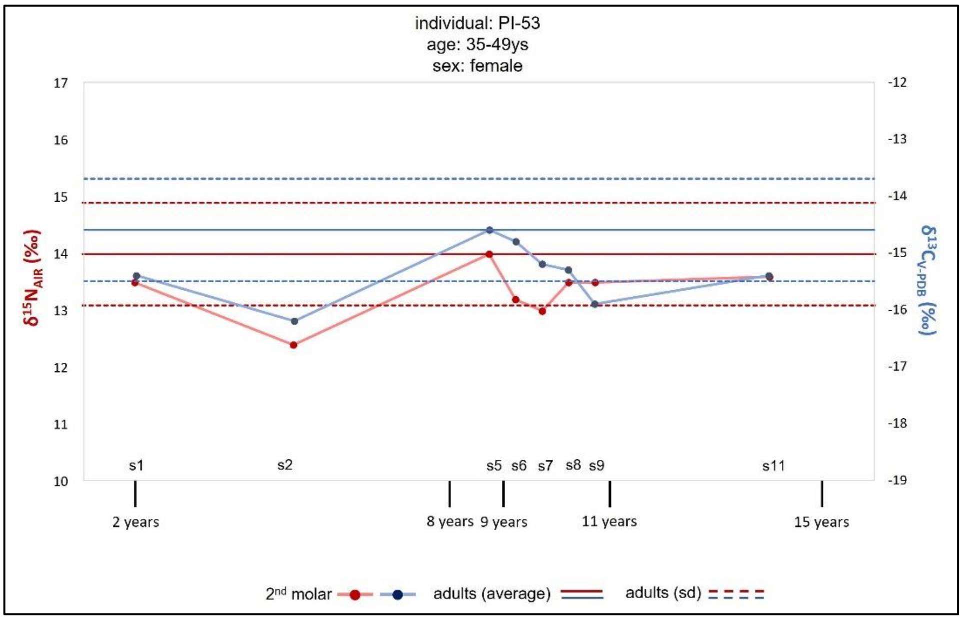

Supplement: S2 File — : Individual dentine δ13C and δ15N isotope ratio profiles for Piaçaguera. (PDF) [file pone.0335680.s002.pdf]
