# Supplementary material for: Dietary variability in Middle Holocene South American shellmounds: Insights from isotopic analysis and an adapted Bayesian MixSIAR model
Source: PLoS One. 2025 Dec 3;20(12):e0335680. doi: 10.1371/journal.pone.0335680 (PMC12674525; doi:10.1371/journal.pone.0335680)

S2 (A-M) Fig: Individual dentine $\delta^{13}C$ (in blue) and $\delta^{15}N$ (in red) isotope ratio profiles for the Moraes series.

Fig S2.A: Individual MO-02-B.

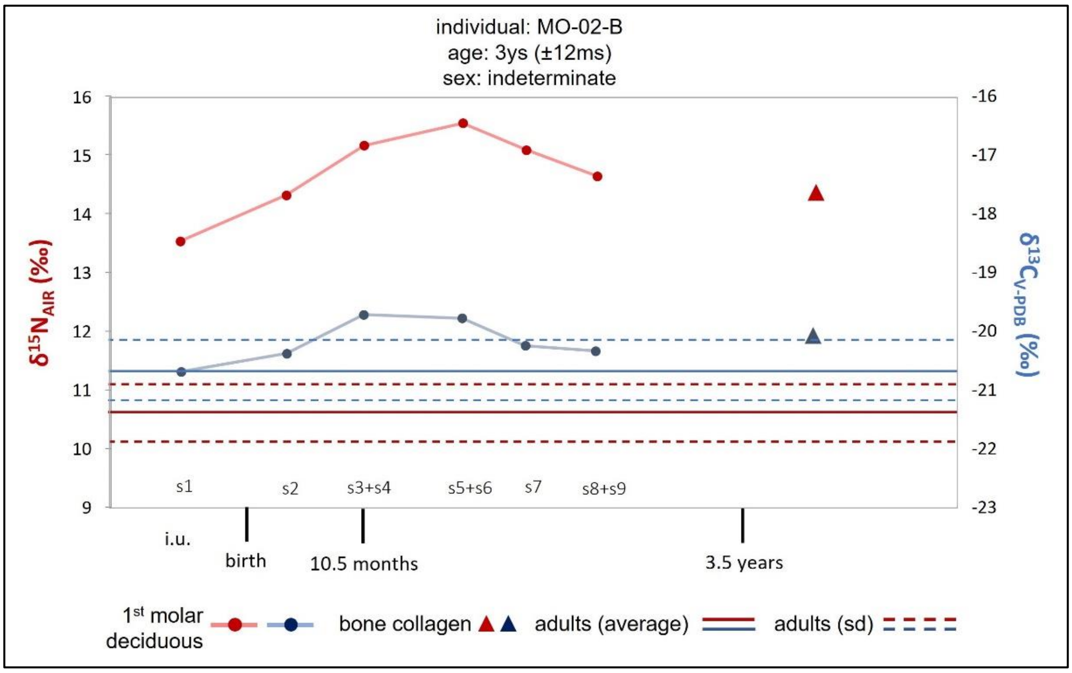

Fig S2.B: Individual MO-04.

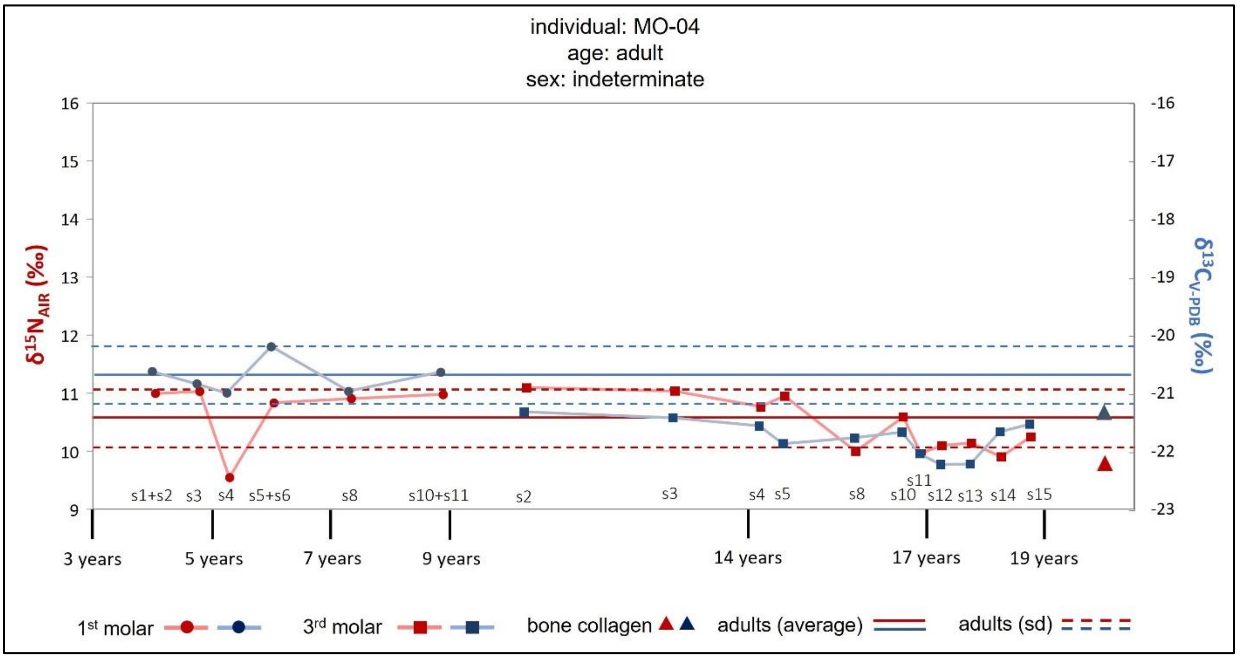

Fig S2.C: Individual MO-06.

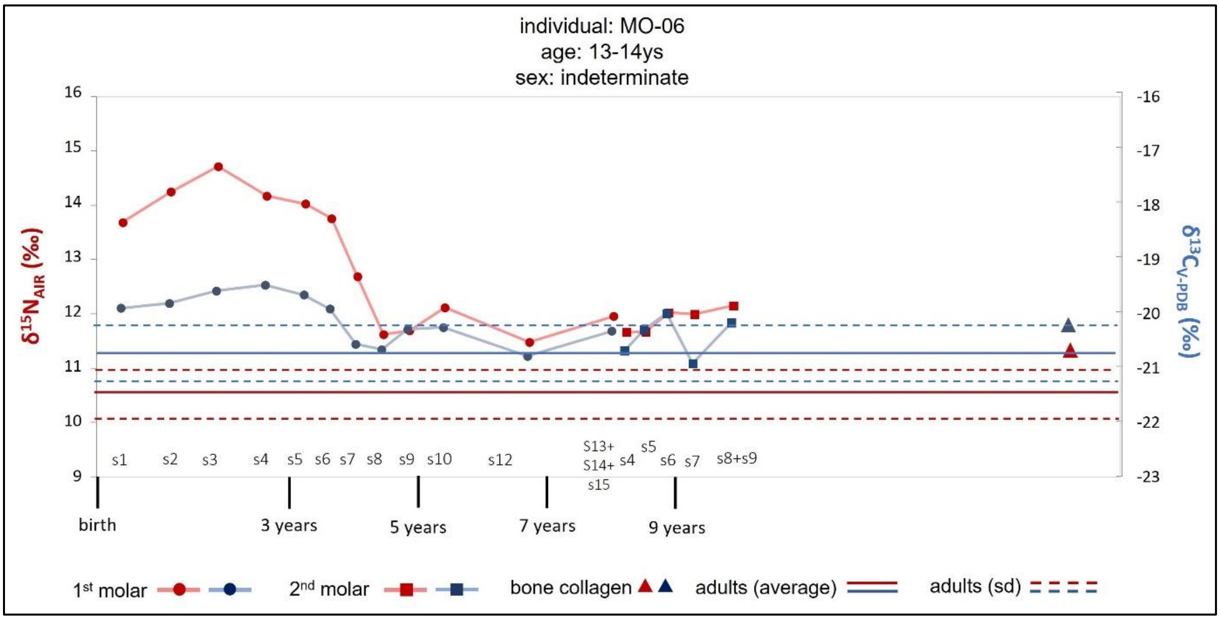

Fig S2.D: Individual MO-09-C.

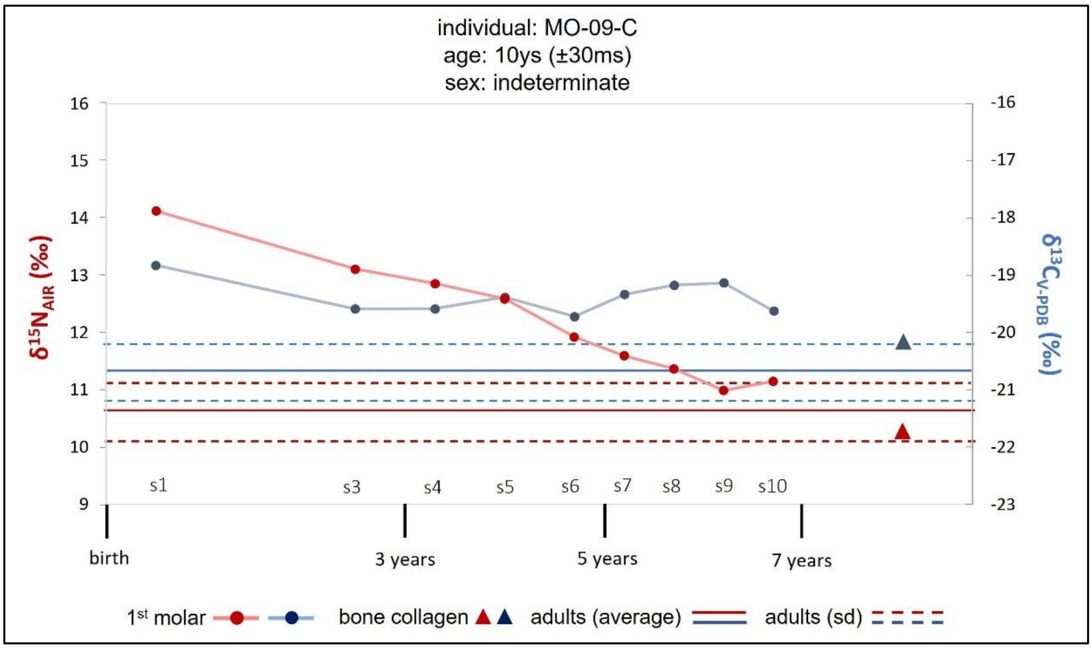

Fig S2.E: Individual MO-12-A.

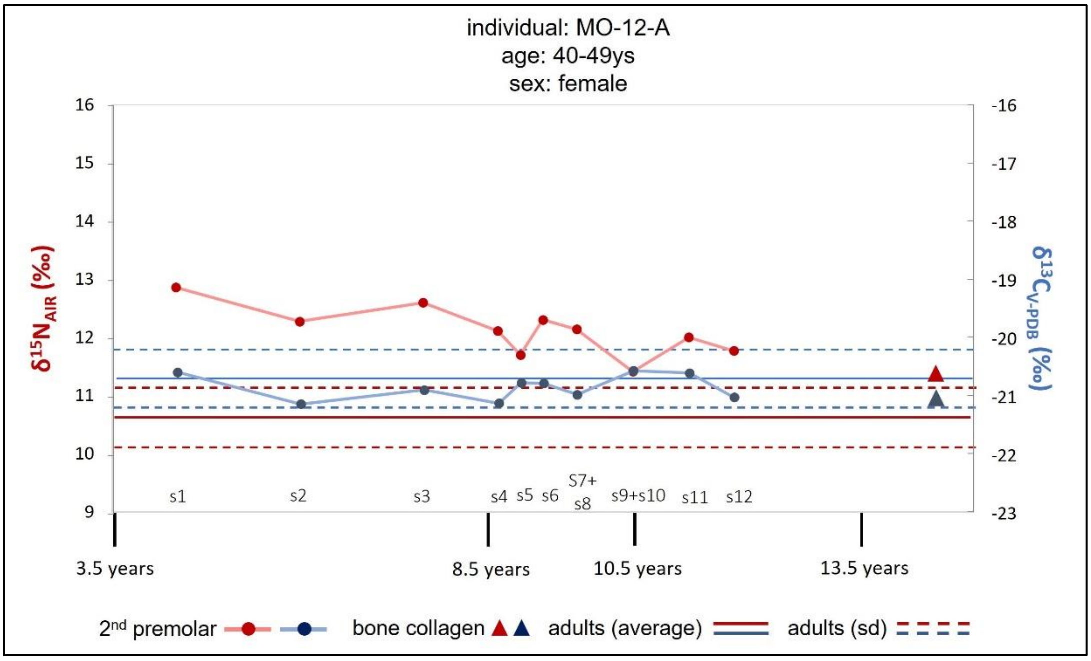

Fig S2.F: Individual MO-21-A.

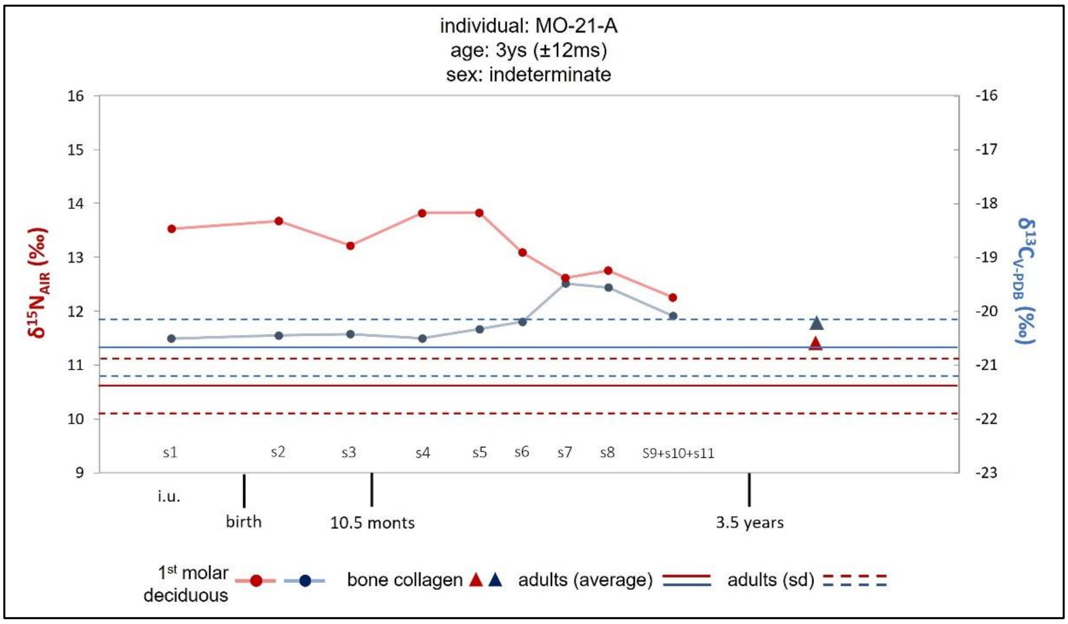

Fig S2.G: Individual MO-27-A.

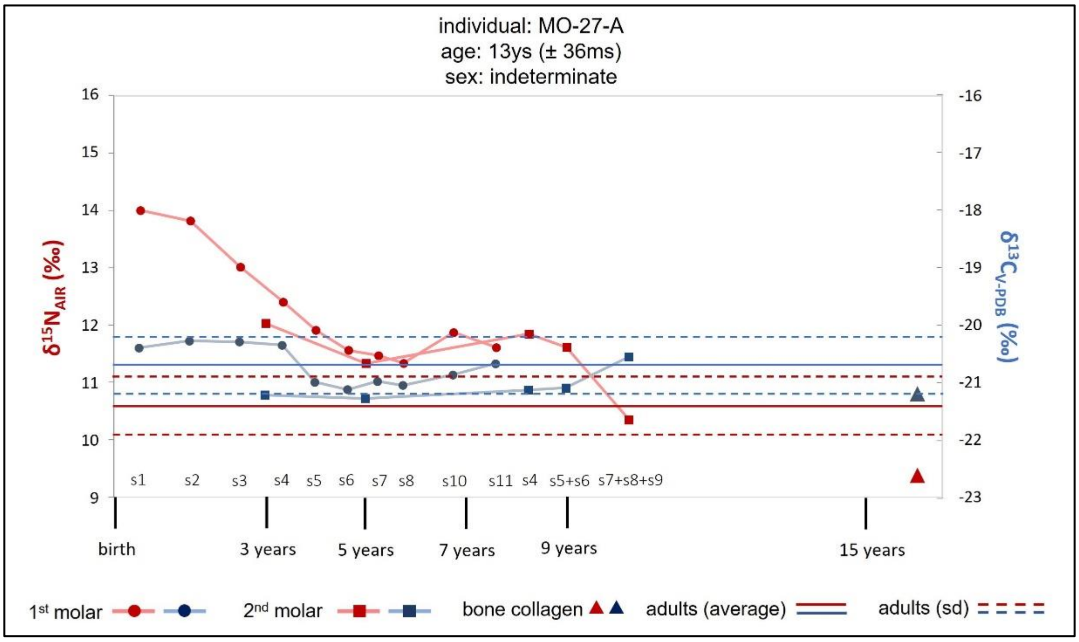

Fig S2.H: Individual MO-31-A.

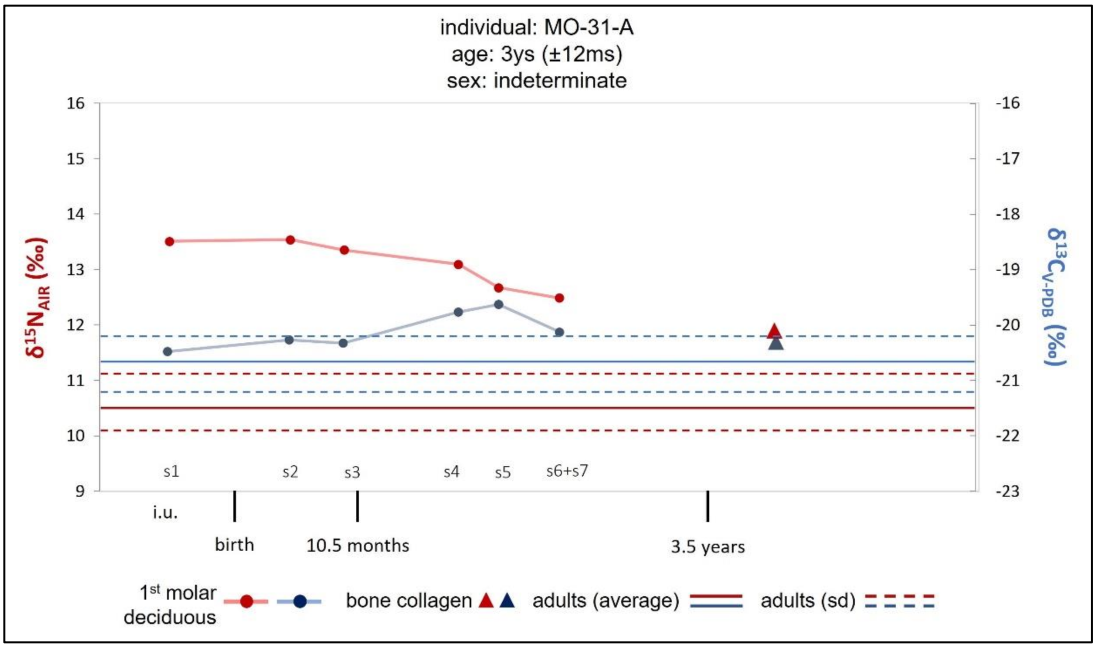

Fig S2.I: Individual MO-38-A.

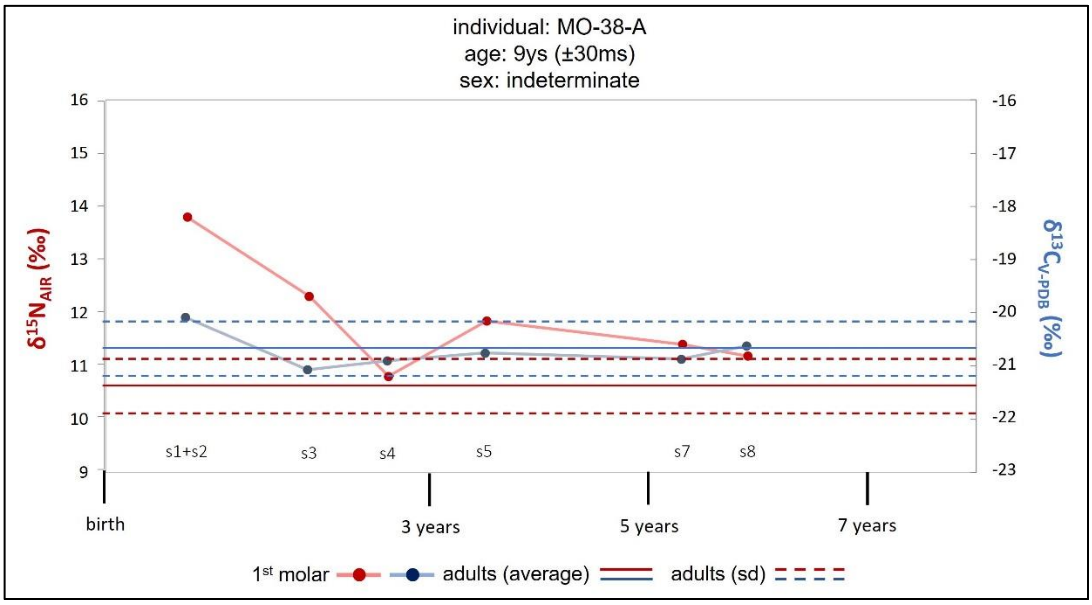

Fig S2.J: Individual MO-41-A.

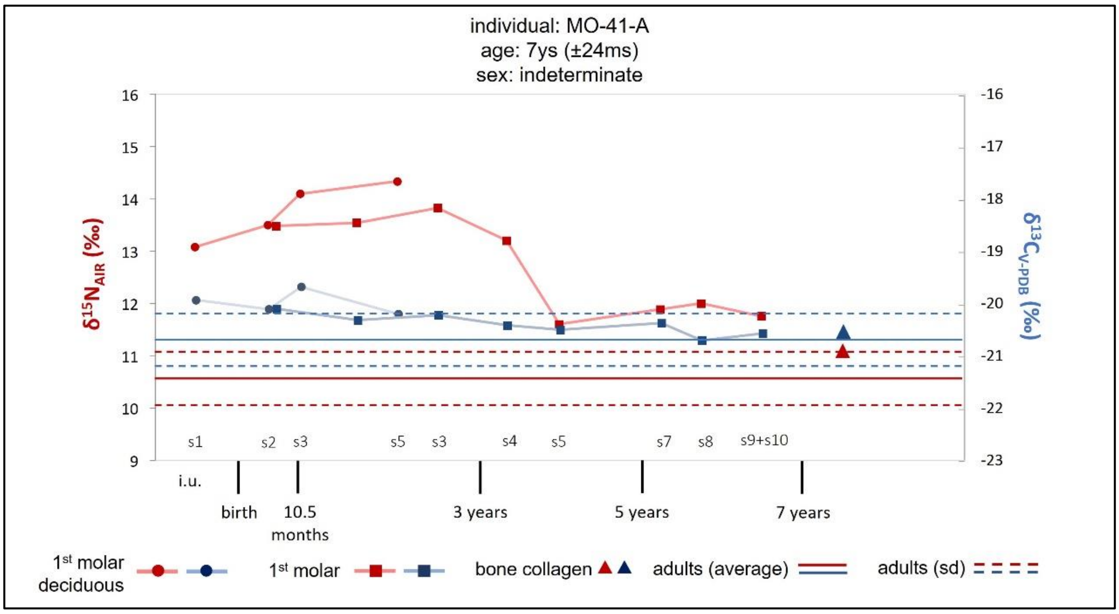

Fig S2.K: Individual MO-41-B.

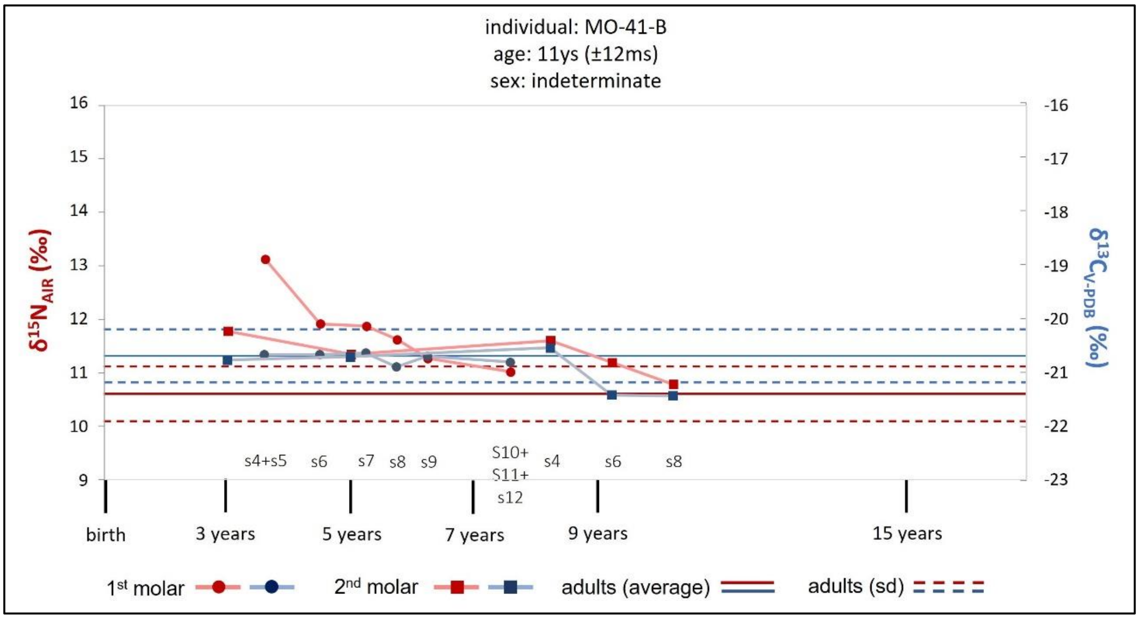

Fig S2.L: Individual MO-42-B.

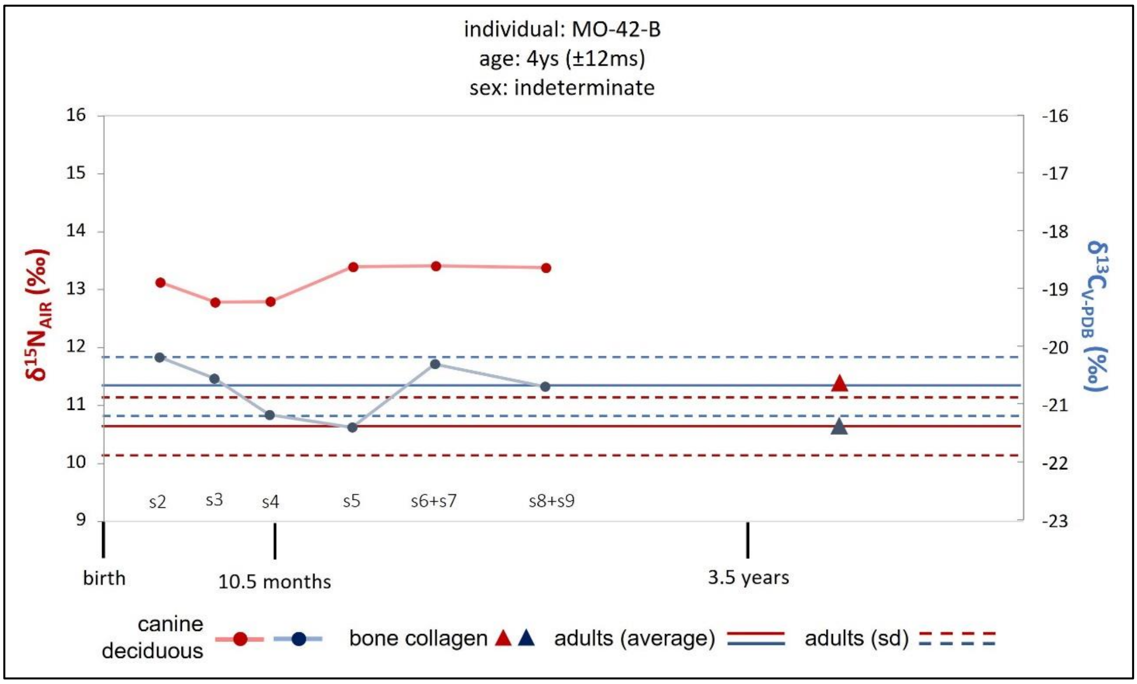

Fig S2.M: Individual MO-43.

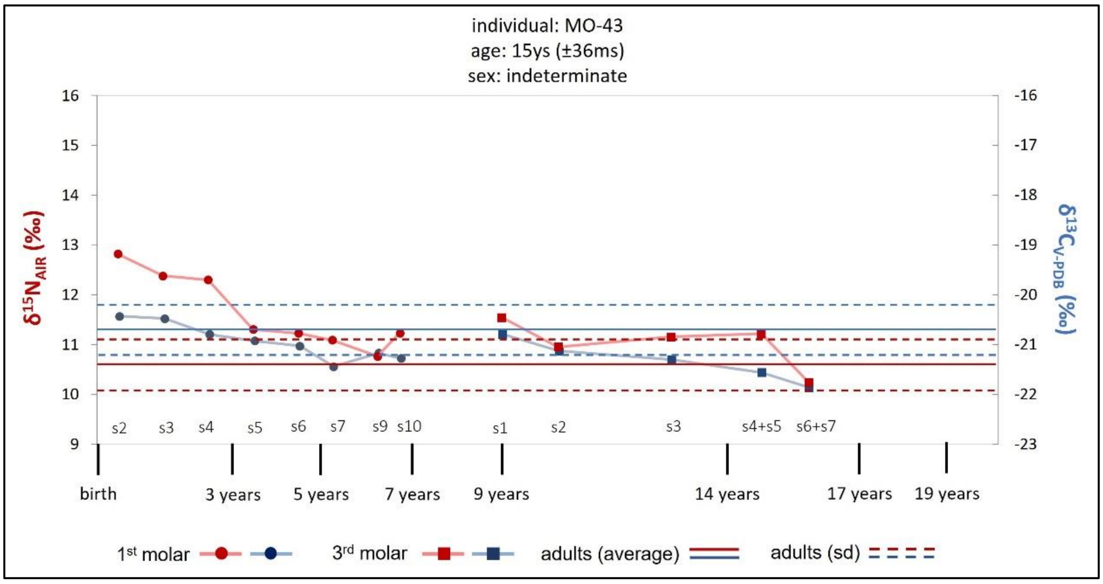

Supplement: S3 File — : Individual dentine δ13C and δ15N isotope ratio profiles for Moraes. (PDF) [file pone.0335680.s003.pdf]
