# Supplementary material for: Dietary variability in Middle Holocene South American shellmounds: Insights from isotopic analysis and an adapted Bayesian MixSIAR model
Source: PLoS One. 2025 Dec 3;20(12):e0335680. doi: 10.1371/journal.pone.0335680 (PMC12674525; doi:10.1371/journal.pone.0335680)
Supplement: S4 File — (PDF) [file pone.0335680.s004.pdf]

S1 file: Normality test

The normality assumption was checked for δ^{13}C and δ^{15}N values from adult bone samples from Piaçaguera and Moraes sites, as "adults" was the only subgroup with independent samples and a sufficiently large sample size. We used Lilliefors' normality test, which is appropriate when testing for normality against unknown parameters that must be estimated from the sample. We rejected normality at the 5% level for δ^{13}C at Moraes ($p < 0.001$), but not for δ^{15}N at Moraes (p = 0.760), nor for δ^{13}C and δ^{15}N at Piaçaguera (p = 0.232 and 0.261, respectively). We also performed Kolmogorov-Smirnov tests with a Lilliefors-like adaptation for the location-scale t-Student family, which generalizes the Normal distribution; in these tests, normality was not reject at the 5% level in any case (Moraes: p = 0.581 and 0.684 for δ^{13}C and δ^{15}N, respectively; Piaçaguera: p = 0.432 and 0.534 for δ^{13}C and δ^{15}N, respectively). We chose to keep the Normal distribution in our model, as the added degree of freedom from the location-scale t-Distribution would increase complexity in a framework that is already underspecified. Instead, we performed a robustness analysis of the model's output excluding the two extreme δ^{13}C values from Moraes (MO-07 and MO-08), which removed the evidence against normality (p = 0.239 and 0.917 for δ^{13}C and δ^{15}N, respectively).

The analysis presented in the manuscript includes the full sample. This Supporting Information contains the results from the adapted MixSIAR model applied to the Moraes site after removing from the analysis two individuals, MO-07 and MO-08, whose extreme δ^{13}C values led to rejection of normality at the 5% level for δ^{13}C values from "adult" age-group. As this supporting material is intended as a robustness check for the conclusions presented in the manuscript, discussions are limited to comparing the model outputs with and without these two observations. Due to the presence of shared estimated parameters across age groups, such as those representing

the tracer values for each food source, removal of samples in the adults age group impacts the model output for all age groups.

For the 5-9 years category, all consumption proportion quartiles change by under one percentage point, and 95% HPD interval limits change by under two percentage points (Fig S1; Table S1). The marine fish, terrestrial animal and C_3 plant 50% HPD interval limits change by under two percentage points as well, while the freshwater fish 50% HPD interval limits show the highest variation – a lower and upper limit 6.5 and 5.5 percentage points higher, respectively. The relative ordering of consumption proportions doesn't change – freshwater fish remains the highest consumption, with terrestrial animals as a close second, C_3 plants as a clear third and marine fish as the lowest.

Figure S1: Posterior density estimates of the distributions of food source group consumption for 5-9 years age-group for Moraes (after exclusion of MO-07 and MO-08), with 50% and 95% probability HPD intervals and the median.

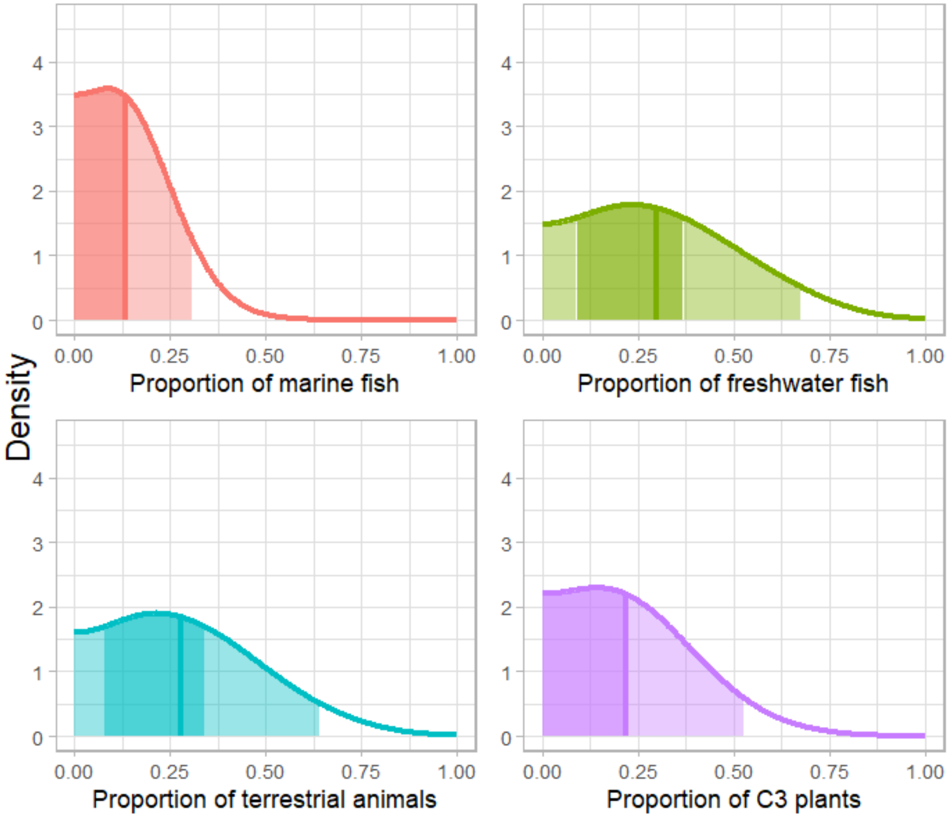

Table S1: 50% and 95% HPD intervals and quartiles of posterior consumption proportions at Moraes (after exclusion of MO-07 and MO-08) for the 5-9 years age group.

		50% Probability HPD Interval		95% Probability HPD Interval				
**Age Group**	**Food Source**	**Inferior Limit**	**Superior Limit**	**Inferior Limit**	**Superior Limit**	**Q1**	**Q2**	**Q3**
5-9 years	Marine fish	0.000	0.134	0.000	0.306	0.081	0.134	0.197
5-9 years	Freshwater fish	0.089	0.369	0.000	0.674	0.163	0.296	0.451
5-9 years	Terrestrial animals	0.079	0.340	0.000	0.641	0.151	0.277	0.421
5-9 years	C3 plants	0.000	0.220	0.000	0.525	0.113	0.218	0.337

For the 9-15 years category, there is little change for the marine fish and freshwater fish consumption proportions (Fig S2; Table S2). For terrestrial animals, there is a decrease of approximately three percentage points for all quartiles, and a corresponding increase between two and three percentage points for all quartiles in C_3 plant consumption. Swings in the 50% HPD interval limits are more pronounced for these two food sources. Overall, the original model output had freshwater fish as the main food source, terrestrial animals as a close second, C_3 plants as a clear third and marine fish as a clear last. The comparison output has freshwater fish still as the first in consumption, with similar estimated values. Terrestrial animals remain as the second but with lower estimated consumption proportions, while C_3 plants remain as the third but with higher estimated values, lowering the gap between the two estimated proportions by approximately six percentage points. Marine fish remain as a clear last.

Figure S2: Posterior density estimates of the distributions of food source group consumption for 9-15 years age-group for Moraes (after exclusion of MO-07 and MO-08), with 50% and 95% probability HPD intervals and the median.

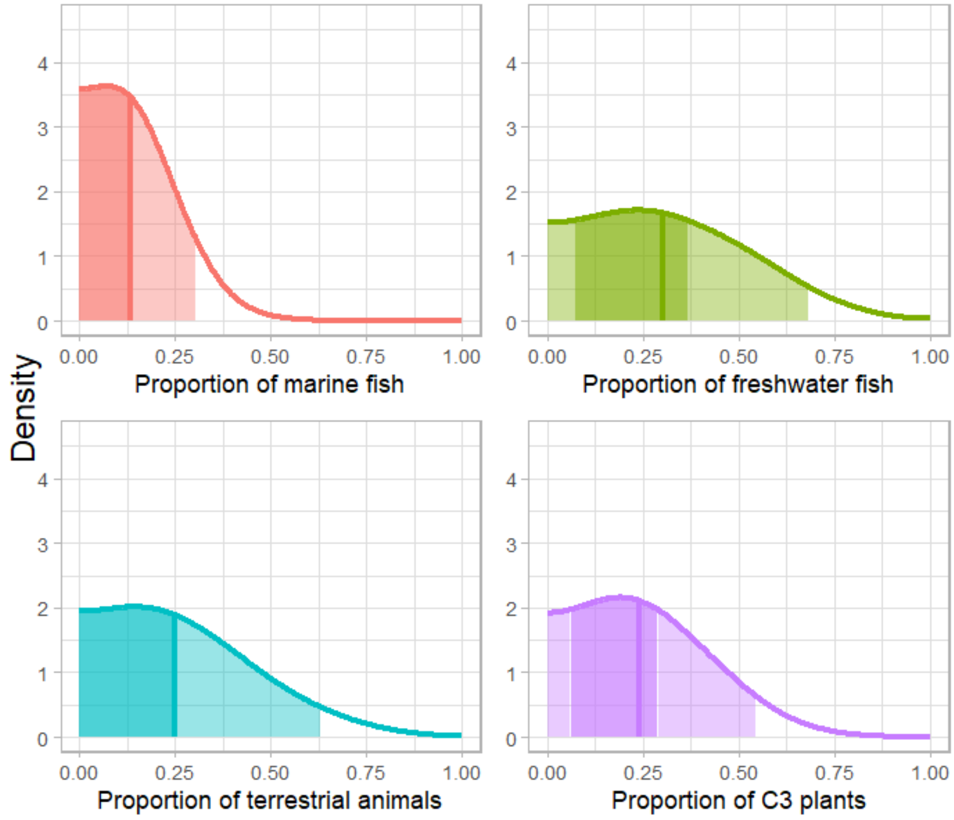

Table S2: 50% and 95% HPD intervals and quartiles of posterior consumption proportions at Moraes (after exclusion of MO-07 and MO-08) for the 9-15 years age group.

		50% Probability HPD Interval		95% Probability HPD Interval				
**Age Group**	**Food Source**	**Inferior Limit**	**Superior Limit**	**Inferior Limit**	**Superior Limit**	**Q1**	**Q2**	**Q3**
9-15 years	Marine fish	0.000	0.132	0.000	0.304	0.078	0.131	0.195
9-15 years	Freshwater fish	0.073	0.367	0.000	0.684	0.162	0.304	0.466
9-15 years	Terrestrial animals	0.000	0.249	0.000	0.629	0.129	0.249	0.396
9-15 years	C3 plants	0.062	0.289	0.000	0.543	0.129	0.238	0.362

For the 15-18 years category, little change is observed in the estimated consumption quartiles, with most changing by under one percentage point and all changing by under two (Fig S3; Table S3). Quartiles for freshwater fish consumption are lowered by approximately one percentage point and for terrestrial animals raised by approximately a point and a half, widening the gap between the two proportions by approximately two and a half percentage points. The 50% HPD interval limits for terrestrial animals show the highest variation, of eight percentage points. Overall, terrestrial animals remain as the food source with the highest consumption proportion, followed closely by freshwater fish, but with a slightly larger gap. C_3 plants and marine fish remain as the third and fourth sources with little change.

Figure S3: Posterior density estimates of the distributions of food source group consumption for 15-18 years age-group for Moraes (after exclusion of MO-07 and MO-08), with 50% and 95% probability HPD intervals and the median.

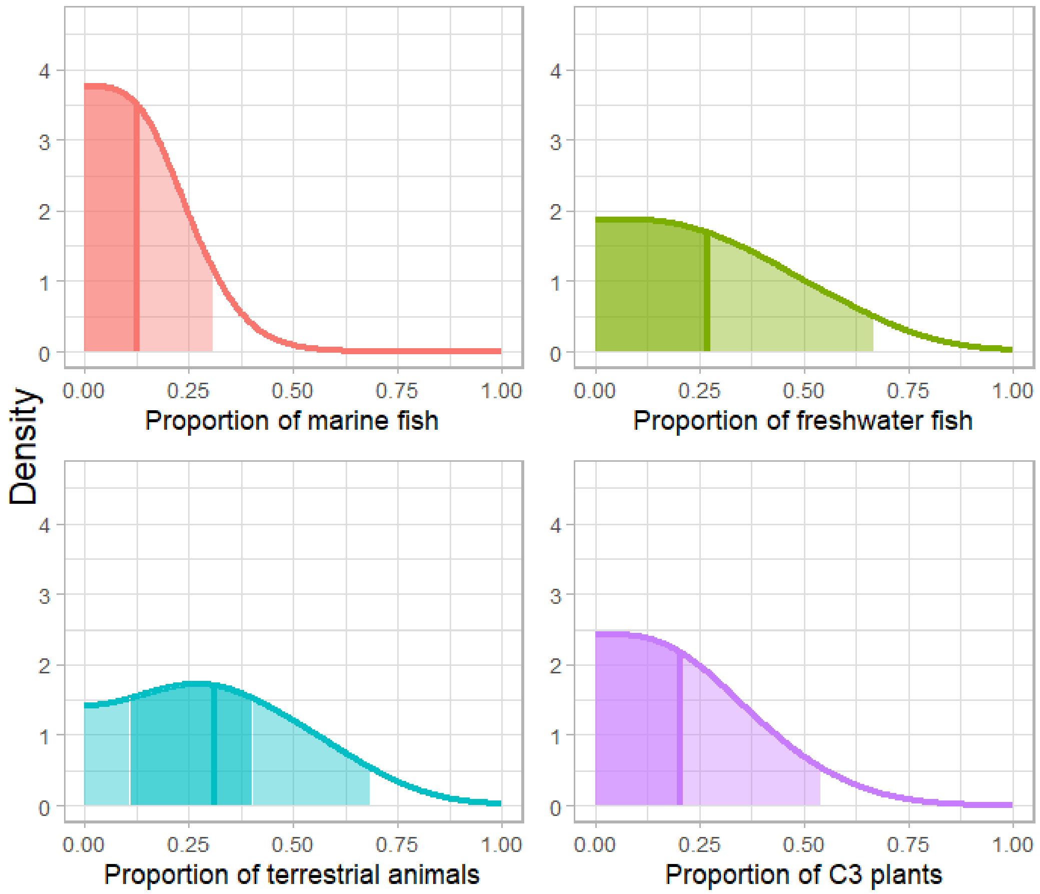

Table S3: 50% and 95% HPD intervals and quartiles of posterior consumption proportions at Moraes (after exclusion of MO-07 and MO-08) for the 15-18 years age group.

		50% Probability HPD Interval		95% Probability HPD Interval				
**Age Group**	**Food Source**	**Inferior Limit**	**Superior Limit**	**Inferior Limit**	**Superior Limit**	**Q1**	**Q2**	**Q3**
15-18 years	Marine fish	0.000	0.126	0.000	0.308	0.072	0.126	0.192
15-18 years	Freshwater fish	0.000	0.271	0.000	0.668	0.133	0.270	0.434
15-18 years	Terrestrial animals	0.109	0.402	0.000	0.684	0.171	0.312	0.468
15-18 years	C3 plants	0.000	0.206	0.000	0.541	0.103	0.205	0.332

For the adults, conclusions are similar to the 15-18 years age group (Fig S4; Table S4). Quartiles for marine fish barely change, for C_3 plants are lowered by approximately one percentage point, for freshwater fish by approximately two and for terrestrial animals are raised by approximately three percentage points. This keeps terrestrial animals as the food source with the highest consumption and freshwater fish as a close second, but with a widened gap, C_3 plants as the clear third and marine fish as the clear last. Again the 50% HPD interval limits for terrestrial animals show the highest variation, of approximately four percentage points on the lower limit and seven on the upper limit. Freshwater fish and C_3 plants 50% HPD interval upper limits are slightly lower.

Figure S4: Posterior density estimates of the distributions of food source group consumption for adult age-group for Moraes (after exclusion of MO-07 and MO-08), with 50% and 95% probability HPD intervals and the median.

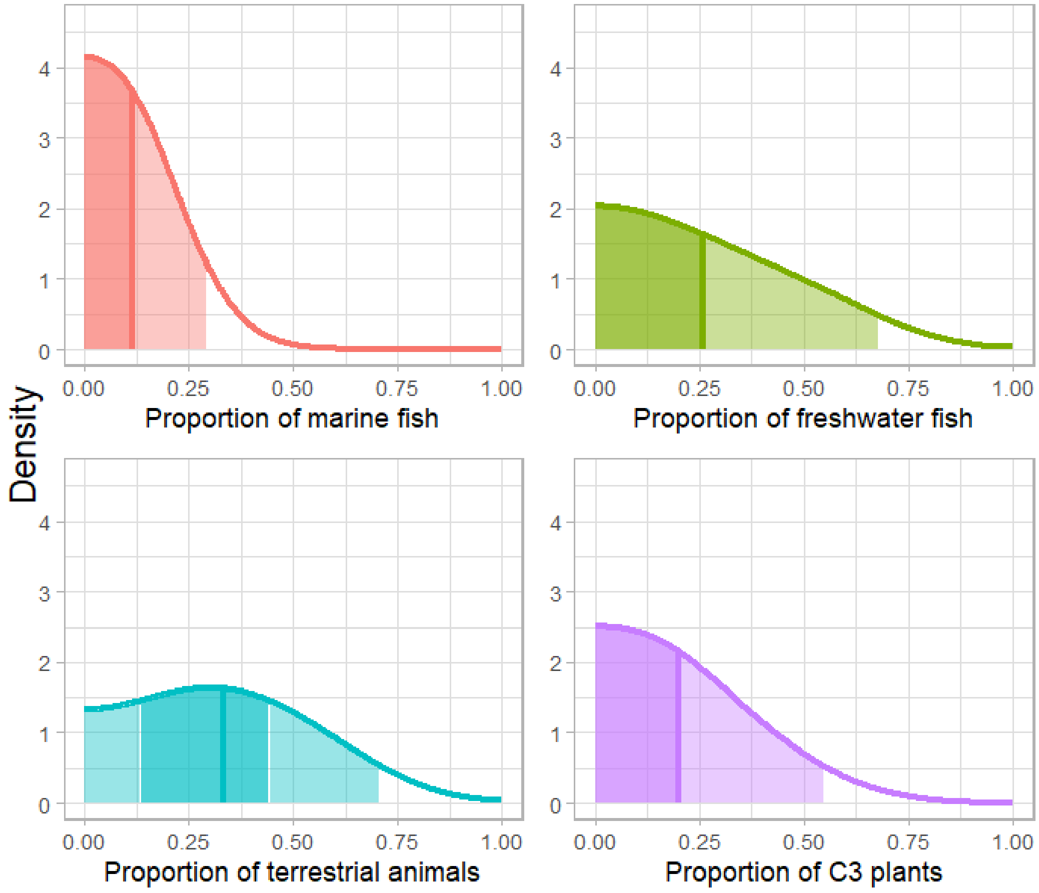

Table S4: 50% and 95% HPD intervals and quartiles of posterior consumption proportions at Moraes (after exclusion of MO-07 and MO-08) for the adult age group.

		50% Probability HPD Interval		95% Probability HPD Interval				
Age Group	Food Source	Inferior Limit	Superior Limit	Inferior Limit	Superior Limit	Q1	Q2	Q3
15-18 years	Marine fish	0.000	0.116	0.000	0.292	0.062	0.115	0.180
15-18 years	Freshwater fish	0.000	0.261	0.000	0.678	0.122	0.259	0.437
15-18 years	Terrestrial animals	0.134	0.443	0.000	0.705	0.182	0.332	0.492
15-18 years	C3 plants	0.000	0.203	0.000	0.549	0.099	0.202	0.335

The model output with removal of the two observations remains consistent with the original conclusions of a diet primarily consisting of terrestrial animals and freshwater fish, with C_3 plants also as a relevant food source and no or very low marine fish consumption. It also remains consistent with decreasing consumption proportions of freshwater fish and increasing consumption proportions of terrestrial animals with increasing age. The model summaries most impacted by the removal of the two observations were the 50% HPD intervals. Graphical analysis of the estimated posterior densities shows the estimated density does not change by much between the two model outputs, but the 50% HPD interval limits are very sensible to a small change in densities that flips the relative ordering of two points. This leads us to consider the posterior distribution quartiles more adequate as model summaries for this comparison between model outputs.
